# Cu and Hydroquinone for the Trifluoromethylation of Unprotected Phenols

**DOI:** 10.1002/ejoc.201801111

**Published:** 2018-10-08

**Authors:** Jakob Pletz, Christoph Koeberl, Michael Fuchs, Oliver Steiner, Walter Goessler, Wolfgang Kroutil

**Affiliations:** ^1^ Institute of Chemistry NAWI Graz BioTechMed Graz University of Graz Heinrichstraße 28 8010 Graz Austria; ^2^ Institute of Chemistry NAWI Graz University of Graz Universitätsplatz 1/1 8010 Graz Austria

**Keywords:** Trifluoromethylation, Phenol, Hydroquinone, Catalysis, Copper

## Abstract

Fluorination and trifluoromethylation are indispensable tools in the preparation of modern pharmaceuticals and APIs. Herein we present a concept for the introduction of a trifluoromethyl group into unprotected phenols employing catalytic copper(I) iodide and hydroquinone, *t*BuOOH, and the Langlois' reagent. The method proceeds under mild conditions and exhibits an extended substrate scope compared to the biocatalytic trifluoromethylation using laccase from *Agaricus bisporus*. Various functional groups such as aldehydes, esters, ethers, ketones and nitriles were tolerated. The hydroquinone‐mediated trifluoromethylation reaction allowed accessing trifluoromethylated phenols, which are cumbersome to prepare via previously known chemical methods.

## Introduction

The introduction of fluoroalkyl‐groups (e.g. CF_3_, CHF_2_, CH_2_F, etc.) into organic molecules has become a major topic in various areas of research, in particular medicinal chemistry and drug discovery^1^, ^2^, ^3^ The presence of highly stable C–F bonds increases metabolic stability, membrane permeability or the binding affinity compared to the non‐fluorinated congeners.^4^ Amongst the fluorine‐containing moieties, the trifluoromethyl group is privileged and trifluoromethylated arenes have been employed in agrochemicals, pharmaceuticals and material science.^5^, ^6^ Despite tremendous success and progress of fluorination chemistry, the selective introduction of trifluoromethyl‐group into organic molecules bearing a phenolic hydroxy group remains challenging.^7^, ^8^, ^9^, ^10^

**Scheme 1:**
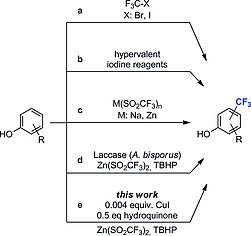
Strategies for the direct trifluoromethylation of free phenols.

Previous research showed that phenols are accepted as substrates, albeit with varied success. Several strategies were used to access these scaffolds by direct trifluoromethylation. Trifluoromethylhalogenides (CF_3_Br, CF_3_I) were used as CF_3_‐radical source with activation by sodium dithionite,^11^ light^12^, ^13^ or H_2_O_2_/Fe^II^ salts (Scheme 1a).^14^, ^15^ These methods gave moderate to low yields for free phenols.^11^, ^12^, ^14^, ^15^ The reaction conditions required – depending on the substrate structure – CF_3_Br pressure,^11^ special choice of Fe^II^ salt^14^ or more advanced instrumentation.^15^ Recently, a light‐enabled redox‐neutral trifluoromethylation was published, which generates the CF_3_‐radical by a Norrish type I concept.^16^ Hypervalent iodine electrophilic trifluoromethylation reagents such as Umemoto's reagent,^17^, ^18^ Togni's reagent,^19^, ^20^, ^21^ and others^18^, ^22^ were employed as well (Scheme 1b). However, few arenes with free phenolic hydroxy groups were accepted as substrates. Examples include hydroquinone (HQ),^17^, ^18^, ^22^ 4‐*tert*‐butylphenol,^19^, ^20^ 2‐napthol^18^ and 2,6‐disubstituted phenols.^21^ The use of the Langlois reagent (NaSO_2_CF_3_) and the corresponding Baran zinc sulfinate [Zn(SO_2_CF_3_)_2_] have found widespread application for the direct radical trifluoromethylation (Scheme 1c). The activation of these reagents is achieved by light‐induced,^23^, ^24^ electrochemical^25^ or peroxide‐mediated^26^ mechanisms. Substrates bearing phenolic hydroxy groups are underrepresented in these studies, presumably due to their oxidative sensitivity. Representative substrates which were trifluoromethylated using trifluoromethylsulfinates are phenol,^24^, ^26^ 2,6‐di‐*tert*‐butylphenol,^23^ 2‐methylbenzo[*d*]thiazol‐5‐ol and quinoxalin‐2‐ol,^25^ albeit with mediocre yields (32–49 %). For free phenols, the Ruppert‐Prakash reagent (TMSCF_3_) in the presence of catalytic silver (AgF) and PhI(OAc)_2_ gave primarily the CF_3_‐ether, while only minor quantities of the aryl‐CF_3_ product were formed.^27^, ^28^, ^29^

We recently reported the first biocatalyst dependent trifluoromethylation of unprotected phenols that employs laccase from *Agaricus bisporus* and trifluoromethylsulfinate salt as the CF_3_‐radical source (Scheme 1d),^29^ giving access to building blocks that are difficult and cumbersome to prepare via other methodologies.^19^, ^20^, ^28^, ^29^, ^30^, ^31^ DFT calculations showed that the regioselectivity of the trifluoromethylation observed was not enzyme controlled,^29^ therefore the question remained what is the role of the protein backbone of the laccase. Laccases perform oxygen activation at the trinuclear Cu center inside the laccase but this was not required in the proposed catalytic cycle. Only the mononuclear type 1 Cu close to the surface seemed to play a major role.^32^, ^33^, ^34^, ^35^, ^36^ Herein we report on the investigation of the role of the copper ion in the laccase catalyzed trifluoromethylation leading to the development of a protein free alternative procedure for trifluoromethylation of phenols (Scheme 1e). The reaction relies on a combination of catalytic CuI and HQ. Initial investigations on the mechanism indicate that it differs from the one involving the biocatalyst.

## Results and Discussion

**Scheme 2:**
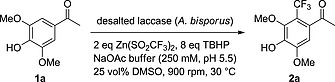
Trifluoromethylation of acetosyringone (**1a**) with desalted laccase solution.

Based on the early reports from Langlois, who used catalytic Cu(SO_2_CF_3_)_2_, NaSO_2_CF_3_ and TBHP for the CF_3_‐radical formation for the trifluoromethylation of arenes, we investigated whether and how free copper ions contribute to the observed reactivity of the laccase in the trifluoromethylation.^26^ The laccase used in the biotransformation was a crude preparation obtained from the *A. bisporus* mushroom. In order to exclude background reactivity by free Cu salts in the enzyme preparation, the laccase solution was desalted via column elution and used directly for the transformation of acetosyringone (**1a**) (Scheme 2).

Interestingly, trifluoromethylation activity was lost and only basal unproductive conversion of substrate **1a** was detected (Table 1, entry 3). This observation might be due to a loss of the reactive mononuclear Cu which may have dissociated during the desalting procedure on the Sephadex material. Analysis of the crude laccase (used for experiment in entry 2) and the desalted laccase solution via ICPMS revealed a depletion in copper concentration of 93 % (see Table SI‐1), which suggests the presence of non‐bound/dissociated copper ions.

**Table 1:** Preliminary experiments leading to the HQ‐mediated trifluoromethylation reaction^a^

Entry	Catalyst	Additive	Conversion [%]^b^	Yield **2a** [%]^b^
1	–	–	10	n.d.
2	laccase^c^	–	97	50
3	desalted laccase^d^	–	15	n.d.
4	CuI^e^	desalted laccase^d^	80	48
5	CuI^e^	–	92	34
6	CuI^e^	HQ^f^	85	48

a 1 equiv. acetosyringone (**1a**, 12.5 µmol), 2 equiv. Zn(SO_2_CF_3_)_2_, 8 equiv. TBHP, 25 vol.‐% DMSO, NaOAc buffer (250 mm, pH 5.5), 30 °C, 900 rpm, 24 h.

b Determined via GC‐FID using 4′‐methoxyacetophenone as internal standard.

c 5 mg mL^–1^ from *A. bisporus*.

d 5 mg mL^–1^ from *A. bisporus*, desalted using a PD MiniTrap G‐25 Sephadex column.

e 0.004 equiv. CuI.

f 0.5 equiv. HQ. conditions.

It might be assumed that the addition of external copper may restore the activity by filling up the vacant sites. Based on the molecular weight of the enzyme (65 kDa),^37^ a suitable Cu concentration was chosen. Indeed, the addition of 200 µm (0.004 equiv.) CuI restored the activity of the desalted laccase solution and a yield of 48 % was detected at 80 % conversion (Table 1, entry 4). The restored laccase activity was confirmed by an activity assay with 2,6‐dimethoxyphenol, in which a drop in trifluoromethylation activity after desalting and a subsequent restoration of activity upon addition of 200 µm CuI was observed (see Table SI‐2). In order to differentiate between the effect of the laccase and the free copper ions, laccase was omitted and 0.004 equiv. CuI were used instead as catalyst (Table 1, entry 5). A conversion of 92 % was detected, similar to the native laccase reaction (97 %, entry 2). In contrast, a significantly decreased yield (34 %) compared to the experiment with the desalted laccase solution and CuI (48 %, entry 4) was observed. Careful experiments were conducted to determine, whether the beneficial effect of the desalted laccase on the CuI‐catalyzed trifluoromethylation was due to unspecific interactions of the copper ions with amino acid residues of the enzyme. The desalted laccase was replaced by bovine serum albumin in the presence of 0.004 equiv. CuI leading to a decreased yield (11 %) and high conversion (86 %). This result implied that the effect of the laccase protein relies on the specific amino acid arrangement at the active site. We hypothesized, that other additives might exist that increase the selectivity of the CuI‐catalyzed trifluoromethylation. Testing the effect of 0.5 equiv. HQ,^38^ the yield of **2a** increased to 48 % (Table 1, entry 6), which correlates well to the values obtained in the laccase‐catalyzed reaction (50 %, entry 2) as well as the desalted laccase with supplemented CuI (48 %, entry 4). It remained unclear, why the selectivity of the trifluoromethylation process is rather low in the laccase‐catalyzed (52 %) and the CuI‐catalyzed, HQ‐mediated reaction (56 %). Alternative reaction products, such as dimers or oligomers, were not identified using TLC, GC–MS or HPLC‐MS.

The CuI‐catalyzed trifluoromethylation reaction turned out to be dependent on the concentration of HQ (Figure SI‐1). Low concentrations of HQ (0.001–0.01 equiv.) had no effect on the conversion while a decrease of conversion was detected at 0.5 equiv. HQ and only half conversion was detected with 2 equiv. HQ.

The highest yield of **2a** was found at a HQ concentration of 0.5 equiv. (53 %). Interestingly, the maximum yield at 0.5 equiv. HQ (53 %) corresponds well with the yield of the optimized laccase‐catalyzed system (50 %, Table 1, entry 2)^29^ In general, HQ only showed a beneficial effect on the product yield in the concentration range between 0.1 equiv. and 1 equiv. HQ.

In the laccase‐catalyzed trifluoromethylation, which exhibits intrinsic enhanced selectivity, HQ showed no beneficial effect (Figure SI‐2). On the contrary, increasing the HQ concentration had a detrimental effect on conversion and yield. For both the Cu‐ and the laccase‐catalyzed reaction, the same amounts of HQ led to similar values of conversion (2 equiv. HQ: 52 % and 54 %) and yield of **2a** (2 equiv.: 25 % and 28 %), respectively (Figure SI‐1 & Figure SI‐2).

Furthermore, it was tested, whether the conversion and hence yield could be increased by altering the CuI‐concentration (Figure SI‐3). Experiments showed, that the trifluoromethylation performed equally well within a CuI concentration range from 0.004 equiv. to 0.1 equiv. (47–50 % yield), whereas stoichiometric amounts of CuI led to substantially decreased yield (15 %). A very low CuI concentration of 0.0001 equiv. CuI gave 56 % conversion and 24 % yield, with only slightly decreased selectivity compared to the reactions with higher CuI‐loading. For further optimizations, the CuI‐concentration was kept at 0.004 equiv.

In the absence of HQ a different behavior concerning the achieved conversion and yield of **2a** at varied copper concentrations was found (Figure SI‐4). Compared to the HQ‐mediated reaction, higher concentrations of CuI are required to obtain high conversions. The highest productivity was observed at 0.01 equiv. CuI (38 % yield) and decreased again at higher concentrations (1 equiv.: 5 % yield). With higher copper concentrations (0.01–1 equiv.), the reaction without HQ run to complete conversion of the starting material **1a** (Figure SI‐4), whereas the HQ‐mediated system reached a plateau at about 90 % conversion (Figure SI‐3). The overall selectivity towards the product **2a** had a maximum of 56 % with HQ but 38 % in the absence.

Various copper salts were compared with Cu^I^ iodide in order to check if conversion and yield depended on the copper species or counterion (Table SI‐3). The results showed, that both Cu^I^ and Cu^II^ salts gave equally high conversions (88–91 %) and comparable yields (40–45 %). It is conceivable, that the initial oxidation state of the catalytic copper salt did not play a role under the oxidative reaction conditions used (TBHP, air). For practical reasons we chose Cu^I^ iodide for our further studies due to the convenient handling of the DMSO stock solutions.

Investigation of the effect of TBHP revealed, that the TBHP concentration needed to be high enough to ensure sufficient CF_3_‐radical formation and that an excess had no significant effect on the yield (Table SI‐4). It was found that 4 equiv. TBHP (entry 4) are needed to reach high conversion of **1a** (91 %, entry 4). Using a larger excess of TBHP, the reaction gave similar yields between 4 equiv. and 10 equiv. (entries 4–7). Since no negative impact of higher TBHP concentrations was observed, the TBHP concentration was kept at 8 equiv. for further studies (entry 6).

An increase in Zn(SO_2_CF_3_)_2_ concentration resulted in increased conversion of the starting material and >99 % conversion was detected with 2.5 equiv. Zn(TFMS)_2_ (Table SI‐5, entry 5). However, the yield showed a maximum of 57 % at 2 equiv. Zn(TFMS)_2_ at 89 % conversion. The concentration of CF_3_‐radicals seems to be both essential for sufficient turnover and promoting the formation of by‐products leading to decreased yields. The high amounts of Zn(SO_2_CF_3_)_2_ needed might be attributed to the unproductive turnover of the CF_3_‐radicals, e.g. forming CF_3_H.^39^ The instability of the Zn(SO_2_CF_3_)_2_ reagent used seemed to be a significant parameter in the absolute yields obtained. Generally, freshly opened containers gave higher yields and repeated opening of the container resulted in a loss of activity, even when flushed with argon and kept at the recommended storage temperature after use.

Investigation of the pH range showed that high conversions and good yields were obtained from pH 5 to pH 7, whereas higher pH values had a deleterious effect on both yield and selectivity of the trifluoromethylation (Table SI‐6). As the pH value did not have a strong impact on the yield, we keep the same pH value as in the biocatalytic trifluoromethylation (pH 5.5) for further optimizations.

Dipolar‐aprotic or dipolar‐protic co‐solvents other than DMSO lead to diminished conversions and yields (Table SI‐7). The trifluoromethylation with 2‐propanol gave the lowest conversion and yield (entry 1), while MeCN and acetone showed higher selectivities towards the product at reduced conversions (entries 2 & 3). As in the laccase‐catalyzed reaction, the highest selectivity was observed with DMSO as a co‐solvent (entry 4).^29^

### Mechanistic Considerations

In order to gain indications for the reaction mechanism, radical scavengers such as TEMPO were tested in the HQ‐mediated trifluoromethylation reaction (Table 2). TEMPO has been described to react with CF_3_‐radicals to give a TEMPO‐CF_3_ adduct, which has been detected via ^19^F‐NMR spectroscopy^40^, ^41^ Addition of 1 equiv. TEMPO to the HQ‐mediated reaction resulted in decreased conversion but at the same time the selectivity towards the product remained high (entry 3). We hypothesize that the TEMPO quenched one equivalent of CF_3_‐radicals [2 equiv. Zn(SO_2_CF_3_)_2_ were used] and did otherwise not interfere with the HQ‐mediated trifluoromethylation reaction. This hypothesis was supported by the results from the reaction with 1.5 equiv. Zn(SO_2_CF_3_)_2_ (Table SI‐5, entry 3), which simulates 0.5 equivalents being quenched by the TEMPO reagent for the scavenger experiment. The conversion (72 %) and yield (34 %) obtained fit nicely with the conversion (69 %) and yield (34 %) for the HQ‐mediated reaction with one equivalent of TEMPO (Table 2, entry 3). A similar behavior was expected for the trifluoromethylation without HQ, namely a reduced turnover due to the scavenging of CF_3_‐radicals. However, without the addition of HQ, TEMPO almost completely inhibited the reaction (entry 4).

**Table 2:** Influence of TEMPO on the HQ‐mediated Cu‐catalyzed trifluoromethylation^a^

Entry	Additive(0.5 equiv.)	Quencher(1 equiv.)	Conversion [%]^b^	Yield **2a** [%]^b^
1	–	–	92	34
2	HQ	–	85	48
3	HQ	TEMPO	69	34
4	–	TEMPO	50	2

a Conditions: 1 equiv. acetosyringone (**1a**, 12.5 µmol), 2 equiv. Zn(SO_2_CF_3_)_2_, 0.004 equiv. CuI, 8 equiv. TBHP, 25 vol.‐% DMSO, NaOAc buffer (250 mm, pH 5.5), 30 °C, 900 rpm, 24 h.

b Determined via GC‐FID using 4′‐methoxyacetophenone as internal standard.

**Scheme 3:**
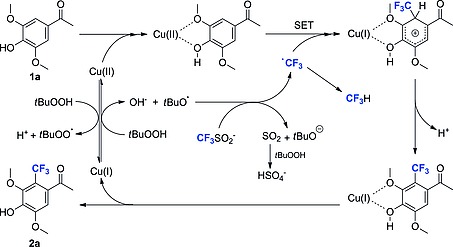
Proposed mechanism of the Cu‐catalyzed trifluoromethylation.

From these experiments, it became evident that both copper salt and TBHP as well as HQ are all crucial for an efficient reaction to take place. Thus, based on our observations and previously described mechanisms by Langlois,^26^ Baran^39^ and Selander,^38^ we propose the following putative radical mechanism (Scheme 3).

The radical process is started by the formation a *tert*‐butoxyl radical from TBHP and a Cu^I^ species, as described previously.^42^, ^43^ Oxidation of the trifluoromethanesulfinate anion by the *tert*‐butoxyl radical gives a CF_3_ radical and SO_2_, which is further oxidized to HSO_4_^–^.^39^, ^44^ Also the formation through an oxidized copper species via a single electron transfer has been suggested.^38^ We suggest that the phenolic substrate **1a** is activated by Cu^II^ via the anisole and/or phenol oxygen atoms. Subsequently, it can be attacked by the CF_3_‐radical via a single electron transfer forming a Cu^I^ complex with a delocalized cationic charge. Deprotonation and dissociation gives the trifluoromethylated product **2a**.

Direct radical trifluoromethylation generally requires an excess of trifluoromethylsulfinate salt, since the CF_3_ radical can engage in unproductive side reactions. The formation of CF_3_H by hydrogen abstraction was already observed by ^19^F‐NMR and the reaction with isobutene generated from TBHP followed by reaction with an arene substrate was suggested.^39^ In the proposed trifluoromethylation mechanism, the unproductive consumption of CF_3_ radicals necessitates the additional formation of *tert*‐butoxyl radicals to drive the generation of CF_3_ radicals. However, according to literature this process requires Cu^I^ which needs to be regenerated by reduction of Cu^II^ with TBHP and concomitant formation of the *tert*‐butyl hydroperoxy radical and H^+^ (Scheme 3).^42^, ^43^, ^45^ The abundance of *tert*‐butyl hydroperoxy and other radical species in the presence of copper salts might be the reason for the overall low selectivity,^45^ presumably promoting multiple side reactions of the reactive phenols or radical intermediates during the single electron transfer process.

A possible role of HQ might be due to the interference of corresponding species [semiquinone radical as well as benzoquinone (BQ)] with other redox partners in the oxidation‐reduction pathways. Upon GC–MS analysis of the reaction mixtures in the presence of HQ, traces of BQ were detected. A similar beneficial effect as observed for HQ was found with BQ (Table 3). BQ and HQ gave identical yields of **2a**, however the selectivity of the HQ‐mediated reaction was slightly higher (entry 2 and entry 3). The laccase‐catalyzed biotransformation already displays a higher selectivity in the absence of these additives and hence the effect of HQ and BQ supplementation was studied. Both HQ and BQ reduced the conversion of the substrate in the biotransformation and only 82 % and 80 % were detected, respectively (entry 5 and entry 6). However, in spite of the decreased reactivity, the selectivity of the biotransformation towards the product remained high with both additives.

**Table 3:** Effect of HQ and BQ on the CuI‐ and laccase‐catalyzed trifluoromethylation reaction^a^

Entry	Catalyst	Additive	Conversion [%]^b^	Yield **2a** [%]^b^
1	CuI	–	92	34
2	CuI	HQ	79	48
3	CuI	BQ	90	47
4	laccase	–	95	41
5	laccase	HQ	82	48
6	laccase	BQ	80	43

a Conditions: catalyst (5 mg mL^–1^ laccase from *A. bisporus* or 0.004 equiv. CuI), 0.5 equiv. additive, 1 equiv. acetosyringone (**1a**, 12.5 µmol), 2 equiv. Zn(SO_2_CF_3_)_2_, 8 equiv. TBHP, 25 vol.‐% DMSO, NaOAc buffer (250 mm, pH 5.5), 30 °C, 900 rpm, 24 h.

b Determined via GC‐FID using 4′‐methoxyacetophenone as internal standard.

A similar rate‐increasing effect of HQ and BQ had already been described in the copper‐catalyzed redox reaction between hydrazine and H_2_O_2_.^46^

Instead of an expected rate‐reduction due to the known radical‐scavenging activities, the authors observed significant rate increases of hydrazine oxidation with the additives HQ, BQ as well as *o*‐phenylenediamine. They proposed that the organic additives are engaged in radical‐generation activities that outrun their radical‐quenching activities. Considering the low concentrations of the additives (100–400 nM) they suggested an influence via a radical‐chain mechanism.^46^ This is in line with our observations for the HQ‐mediated trifluoromethylation, as a HQ concentration of 0.1 equiv. has already a pronounced beneficial effect on the reaction (Figure SI‐1). The inhibiting effect of higher HQ concentrations might be ascribed to an imbalance in the previously mentioned radical‐generation and radical‐quenching activities.

In another communication, the addition of a catalytic amount of BQ (0.2 equiv.) was found to be beneficial for the PhI(OAc)_2_‐mediated oxidative trifluoromethylation of arenes with CF_3_SiMe_3_ under metal‐free conditions.^47^ A stoichiometric amount of BQ on the other hand led to inhibition of the reaction but the exact role of BQ remained unclear. The authors tentatively suggest a radical‐free mechanism, which has been proposed by Sanford and Bräse in Ag‐mediated trifluoromethylations.^48^, ^49^

**Scheme 4:**
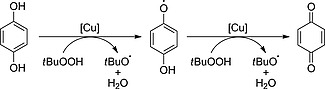
Proposed Cu‐catalyzed formation of *t*BuO‐radicals in the presence of HQ or the semiquinone radical.

The promoting effect of HQ in the trifluoromethylation of phenols might be ascribed to the formation of *t*BuO‐radicals (Scheme 4). The reaction is similar to a proposed mechanism in the copper‐catalyzed, H_2_O_2_‐mediated oxidation of hydrazine.^46^ In the presence of copper ions, *t*BuOOH oxidizes HQ to the semiquinone radical releasing a *tert*‐butoxyl radical and water. The same process happens again to give BQ and a second *tert*‐butoxyl radical. It may be hypothesized, that this formation of *tert*‐butoxyl radicals leads to a higher CF_3_ radical concentration, increasing the initial reaction rate until consumption of HQ and semiquinone. For this initial period, there would be more *tert*‐butoxyl radicals and less *tert*‐butyl hydroperoxy radicals formed, which might lead to less side reactions and a higher selectivity.

**Figure 1:**
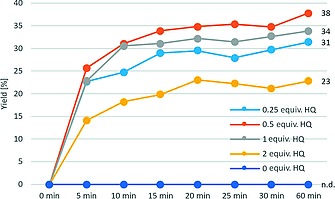
Short‐time kinetics of the Cu‐catalyzed trifluoromethylation at varied amounts of HQ. Conditions: 1 equiv. acetosyringone (**1a**, 25.0 µmol), 2 equiv. Zn(SO_2_CF_3_)_2_, 0.004 equiv. CuI, 8 equiv. TBHP, 25 vol.‐% DMSO, NaOAc buffer (250 mm, pH 5.5), 30 °C, 900 rpm.

This is in line with the observed kinetic behavior of the HQ‐mediated trifluoromethylation reaction (Figure 1). HQ concentrations close to the most productive concentration of 0.5 equiv. were studied (see Figure SI‐1) and significant rate accelerations for all concentrations (0.25 equiv., 0.5 equiv., 1 equiv. and 2 equiv.) were observed. The presence of HQ seems to affect the initial reaction rate directly and after 5 min similar yields were found for the reactions with 0.25 equiv. (23 %), 0.5 equiv. (26 %) and 1 equiv. HQ (23 %). 2 equiv. HQ noticeably decreased the initial rate and a lower yield (14 %) was detected. In comparison, the reaction without HQ did not give any conversion in the investigated time frame (60 min). For all reactions, the reaction rate slows down after 15 min and only sluggishly proceeds to the final product titers after 24 h (0 equiv. HQ: 34 %, 0.25 equiv.: 50 %, 0.5 equiv.: 47 %, 1 equiv.: 40 %, 2 equiv.: 26 %). GC–MS of the reaction mixtures with 1 equiv. and 2 equiv. HQ after 15 min indicated traces of a by‐product with the mass of the HQ with a CF_3_ group attached.

GC‐FID indicated full consumption of HQ after 5 min (0.25 equiv., 0.5 equiv., 1 equiv.), whereas 2 equiv. of HQ were only converted after 10 min. For all reactions, the reaction rate after the first 15 min very much resembles the slow, HQ‐free CuI‐catalyzed trifluoromethylation reaction. These observations suggest that the beneficial effect of HQ lasts only shortly and precedes the normal, much slower, CuI‐catalyzed trifluoromethylation reaction. However, attempts to reinvigorate the reaction after 15 min by addition of another portion of Zn(SO_2_CF_3_)_2_, HQ and TBHP did not lead to higher yields and/or selectivities.

### Regioselectivity and Functional Group Tolerance

To tap the scope and functional group tolerance of this protocol various phenols were transformed under optimized conditions (Table 4, entries 1–6). If the *ortho*‐ and *para*‐positions of the phenol were blocked in substrates **1a** and **1b**, the products **2a** and **2b** with the CF_3_‐moiety in *meta*‐position were isolated with good regio‐control (entry 1 and 2). The transformation of substrate **1c**, bearing only one substituent *ortho* to the phenolic hydroxy group, led to a mixture of regioisomers, albeit with significant preference for the *meta*‐isomer **2c** (*major/minor1/minor2* = 17:2:1, entry 3). The ratio of regioisomers formed in the synthesis of **2c** was similar to the mixture obtained in the biotransformation (*major/minor1/minor2* = 10:1:1, entry 3).^29^ The product of the initial trifluoromethylation process was more reactive under these conditions than in the biotransformation and 5 % of a bis‐trifluoromethylated product was detected (entry 3). The GC–MS spectra of the reaction mixtures with **1a** and **1c** (entry 1 and 3) show traces of by‐products with identical mass as the desired product but different fragmentation patterns, presumably the *O*‐trifluoromethylated congeners (entry 1 and 3). Interestingly, the anisole substrate **1d** reacted under the HQ‐mediated trifluoromethylation conditions, whereas the laccase‐catalyzed trifluoromethylation did not accept substrates devoid of phenolic hydroxy groups (entry 4), thus the here presented procedure allowed to extend the substrate scope. The transformation of **1d** led to a mixture of regioisomers with a preference for isomer **2d** (*major/minor* = 11:1, entry 4) and 26 % of unreacted substrate. The phenol **1e**, devoid of *ortho*‐methoxy substituents, gave exclusively the product **2e** bearing the CF_3_‐group *ortho* to the phenolic hydroxy group with 19 % isolated yield (entry 5). Substrate **1f**, possessing a nitrile‐ instead of an acetyl group in *para*‐position, led to an inseparable mixture of the *ortho*‐ and *meta*‐isomer (4:1, entry 6). In contrast, the biocatalytic trifluoromethylation gave exclusively the *ortho*‐isomer **2f**.^29^

**Table 4:**
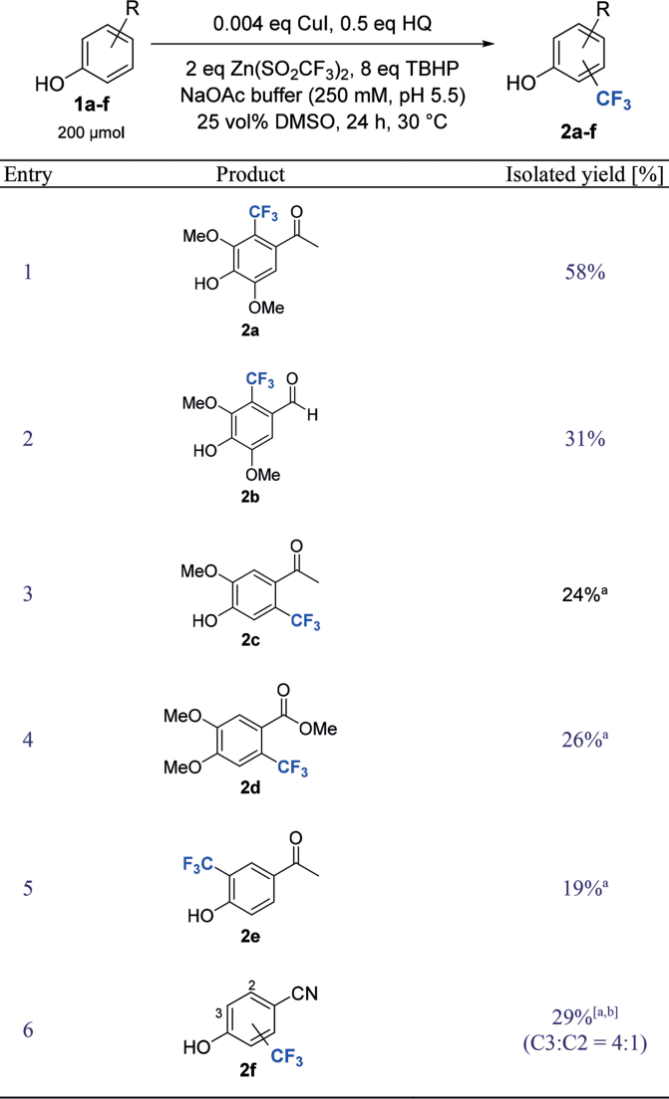
Preparative HQ‐mediated trifluoromethylation (0.2 mmol) of free phenols

48 h reaction time.

Overall isolated ield of both regioisomers.

Similar to the laccase‐catalyzed trifluoromethylation, the HQ‐mediated reaction tolerated aldehyde‐, ester‐, ketone‐ and nitrile‐functionalities. As previously reported, literature methods for the trifluoromethylation gave highly diminished yields with free phenols compared to the laccase‐catalyzed reaction.^29^ The simple and mild HQ‐mediated CuI‐catalyzed trifluoromethylation gives very similar yields and selectivities as the biocatalytic reaction with the commercially available laccase from *Agaricus bisporus*.

## Conclusions

A protein free procedure requiring only 0.004 equivalents of CuI is reported for the direct radical trifluoromethylation of free phenols as well as methoxy substituted benzene at mild conditions (30 °C) and a substrate scope encompassing as substituent ketone, aldehyde, ester or nitrile. In this protocol, the additive HQ enabled an increased reaction rate compared to a reaction in the absence of HQ, presumably by an accelerated CF_3_‐radical formation pathway. Furthermore, the addition of HQ led to a significant higher yield of the products. The here presented protocol is not limited to phenolic hydroxy group, which was a prerequisite for laccase‐catalyzed trifluoromethylation, thus it represents an extension to the enzymatic options.

## Experimental Section

All starting materials were purchased from commercial suppliers and used as received unless stated otherwise. TBHP was obtained as 70 wt.‐% in water from Sigma Aldrich. Laccase originating from *Agaricus bisporus* (>4 U mg^–1^) was bought as a freeze dried powder from Sigma Aldrich and used as received. Analytical thin layer chromatography (TLC) was carried out on Merck TLC silica gel aluminum sheets (silica gel 60, F_254_, 20 × 20 cm) and spots were visualized by UV light (*λ* = 254 nm) and by staining with cerium ammonium molybdate solution and developed by heating with a heat gun. ^1^H‐, ^13^C‐ and ^19^F‐NMR spectra were recorded on a Bruker AVANCE III 300 spectrometer (^1^H: 300.36 MHz; ^13^C: 75.53 MHz, ^19^F: 282 MHz) with an autosampler and a Varian Unity Inova 500 spectrometer (^1^H: 499.88 MHz; ^13^C: 125.69 MHz). Chemical shifts were referenced to the residual proton and carbon signal of the deuterated solvent [CDCl_3_: *δ* = 7.260 ppm (^1^H), 77.160 ppm (^13^C); [D_4_]MeOH: *δ* = 3.31 (^1^H), 49.00 ppm (^13^C), [D_6_]acetone: *δ* = 29.84 (^13^C)]. Chemical shifts *δ* are given in ppm (parts per million) and coupling constants *J* in Hz (Hertz). Signal multiplicities are abbreviated as s (singlet), bs (broad singlet), d (doublet), dd (doublet of doublet), t (triplet) and m (multiplet). Deuterated solvents for nuclear resonance spectroscopy were purchased from Roth, Armar Chemicals and Euriso‐top®. GC‐FID measurements were performed on an Agilent 7890A GC system, equipped with an FID detector and a HP‐5 column (30 m × 0.32 mm × 0.25 µm film) using He at a total flow rate of 35 mL min^–1^. Temperature program: 100 °C, hold 1 min, 20 °C min^–1^ 300 °C, hold 1 min, inlet temperature 300 °C, split ratio 15:1. GC–MS measurements were performed on an Agilent 7890A GC system, equipped with an Agilent 5975C mass‐selective detector (EI 70 eV) and a HP‐5‐MS column (30 m × 0.25 mm × 0.25 µm film) using He at a flow rate of 0.5 mL min^–1^. Temperature program: 100 °C, hold 0.5 min, 10 °C min^–1^ 300 °C, hold 2 min, inlet temperature 250 °C. Purification via preparative reversed‐phase HPLC was performed on a Thermo Scientific Dionex UltiMate 3000 system with UltiMate 3000 pump, UltiMate 3000 autosampler, UltiMate 3000 column compartment, UltiMate 3000 diode array detector (deuterium lamp, *λ* = 190 –380 nm) and a UltiMate 3000 automatic fraction collector. The corresponding compound mixtures were purified on a RP Macherey–Nagel 125/21 Nucleodur® 100–5 C18ec column (21 × 125 mm, 5.0 µm). Signals were detected at 210 nm and 254 nm. Degassed acetonitrile (Chem. Lab NV, HPLC grade) with 0.1 vol.‐% formic acid and degassed water (Barnstead NANOpure®, ultrapure water system) with 0.1 vol.‐% formic acid were used as mobile phase. The gradients for the purification of products in the preparative scale reactions are given in the respective experimental procedures. Melting points were determined on a Mel‐Temp® melting point apparatus from Electrothermal with an integrated microscopical support. They were measured in open capillary tubes with a mercury‐in‐glass thermometer and were not corrected. IR spectra were recorded neat on a Bruker Alpha‐P (ATR) instrument. High resolution mass spectra were recorded on an Agilent 6230 TOF LC/MS using ESI method (positive mode, nozzle voltage 2.0 kV) with coupled Agilent 1260 Infinity Series HPLC. For the copper determination via ICPMS samples were diluted (1+29) with ultrapure water (18.2 MΩ cm). Copper concentrations were determined with an Agilent inductively coupled plasma mass spectrometer (Agilent 8800 or 7700x, Agilent Technologies, Waldbronn, Germany) at a mass to‐charge ratio of 65 using Helium as collision gas. An external calibration with concentrations of 0.1, 0.5, 1.0, 5.0, 10 and 50 µg Cu L^–1^ were prepared from a copper stock standard solution (Single element ICP‐standard, 1000 mg mL^–1^, Art.No 2426.1, Carl Roth GmbH, Karlsruhe, Germany). Germanium at *m/z* 74 served as internal standard to correct for possible matrix effects. The trueness of the measurement was checked with the NIST SRM 1640a (trace elements in water). The obtained result (89.0 µg Cu L^–1^) agreed well with the certified concentration (85.75 ± 0.51 µg Cu L^–1^).

**HQ‐Mediated Cu‐Catalyzed Trifluoromethylation on Preparative Scale:** A 15 mL Sarstedt tube was charged with the phenol/anisole (200 µmol, 1 equiv.), hydroquinone (100 µmol, 0.5 equiv.), Zn(SO_2_CF_3_)**·**2H_2_O (400 µmol, 2 equiv.) and 848 µL DMSO. The mixture was vortexed and placed into an ultrasonic bath until a homogeneous solution was obtained. CuI (800 nmol, 0.004 equiv.) was added as a stock solution (160 µL, 1.5 mg CuI in 2.00 mL DMSO) followed by NaOAc buffer (2.8 mL, 250 mm, pH 5.5) and the mixture was thoroughly mixed. TBHP (70 % in H_2_O, 1.60 mmol, 8 equiv.) was added in one portion, the tube was sealed, horizontally placed into an orbital shaker and was shaken at 500 rpm at 30 °C for the time specified in the respective procedures. EtOAc (6 mL) was added and the mixture was thoroughly mixed (vortex). The phases were separated and the aqueous layer was extracted with EtOAc (4 × 6 mL). The combined organic phase was dried with Na_2_SO_4_, filtered and the solvent was removed on the rotary evaporator. The product was purified by preparative reversed‐phase HPLC using the gradients specified in the respective procedures.

**1‐[4‐Hydroxy‐3,5‐dimethoxy‐2‐(trifluoromethyl)phenyl]ethan‐1‐one (2a):** 39.2 mg (200 µmol, 1 equiv.) acetosyringone (**1a**), 24 h reaction time, purification via preparative reversed‐phase chromatography. Method: column oven 30 °C, flow rate 14 mL/min; 0.0–5.0 min MeCN/H_2_O = 3:97 (v/v), 5.0–30.0 min linear increase to MeCN/H_2_O = 55:45 (v/v), 30.0–31.0 min linear increase to MeCN/H_2_O = 100:0 (v/v), 31.0 min–36.0 min hold MeCN/H_2_O = 100:0 (v/v). Yield: 30.6 mg (116 µmol, 58 %) off‐white solid; *R*_f_ = 0.38 (cyclohexane/EtOAc, 1:1); m.p. 114–115 °C; ^1^H NMR (300.13 MHz, CDCl_3_): *δ* = 6.50 (s, 1 H, CH_arom_), 5.83 (s, 1 H, OH), 3.96 (s, 3 H, OCH_3_), 3.93 (s, 3 H, OCH_3_), 2.58–2.37 ppm (m, 3 H, CH_3_); ^13^C NMR (75.47 MHz, CDCl_3_): *δ* = 202.7 (C_q_, C=O), 150.0 (C_q_), 145.9 [C_q_, q, ^3^ *J*(C,F) = 2.0 Hz], 140.2 (C_q_), 133.8 [C_q_, q, ^3^ *J*(C,F) = 2.5 Hz], 123.7 [q, ^1^ *J*(C,F) = 273.4 Hz, C_q_, CF_3_], 113.6 [C_q_, q, ^2^ *J*(C,F) = 31.0 Hz], 103.8 [CH_arom_, the *J*(C,F) coupling was not resolved on the 300 MHz NMR instrument], 61.5 (OCH_3_), 56.6 (OCH_3_), 31.6 ppm [q, ^4^ *J*(C,F) = 3.2 Hz, CH_3_]; ^19^F NMR (282 MHz, CDCl_3_): *δ* = –54.7 ppm. MS (70 eV): *m/z* (%): 264 [M^+^] (51), 250 (11), 249 (100), 245 (7), 229 (25), 201 (8), 186 (7), 160 (6), 115 (5), 77 (5), 43 (40); the spectra are in accordance with previously reported data.^29^

**4‐Hydroxy‐3,5‐dimethoxy‐2‐(trifluoromethyl)benzaldehyde (2b):** 37.2 mg (200 µmol, 1 equiv.) 4‐hydroxy‐3,5‐dimethoxybenzaldehyde (**1b**), 24 h reaction time, purification via preparative reversed‐phase chromatography. Method: column oven 30 °C, flow rate 14 mL/min; 0.0–5.0 min MeCN/H_2_O = 3:97 (v/v), 5.0–30.0 min linear increase to MeCN/H_2_O = 60:40 (v/v), 30.0–31.0 min linear increase to MeCN/H_2_O = 100:0 (v/v), 31.0 min–36.0 min hold MeCN/H_2_O = 100:0 (v/v). Yield: 15.7 mg (62.8 µmol, 31 %) yellow solid; *R*_f_ = 0.47 (cyclohexane/EtOAc, 1:1), m.p. 108–111 °C, ^1^H NMR (300.13 MHz, CDCl_3_): *δ* = 10.29 [q, ^4^ *J*(H,F) = 2.3 Hz, 1 H, CHO], 7.40 (s, 1 H, CH_arom_), 6.16 (br. s, 1 H, OH), 4.00 (s, 3 H, OCH_3_), 3.96 ppm (s, 3 H, OCH_3_); ^13^C NMR (75.47 MHz, CDCl_3_): *δ* = 189.0 [q, ^4^ *J*(C,F) = 6.0 Hz, CHO], 149.6 (C_q_), 145.9 [C_q_, q, ^3^ *J*(C,F) = 2.5 Hz], 144.4 (C_q_), 127.5 [C_q_, q, ^3^ *J*(C,F) = 1.0 Hz], 124.3 [q, ^1^ *J*(C,F) = 275.5 Hz, CF_3_], 118.9 [q, ^1^ *J*(C,F) = 31.4 Hz, C_q_, the two lower signals of the quadruplet were not detected due to low intensity], 106.4 (CH_arom_), 62.0 (OCH_3_), 56.7 ppm (OCH_3_); ^19^F NMR (282 MHz, CDCl_3_): *δ* = –50.63 ppm (d, *J* = 2.3 Hz), the spectra are in accordance with previously reported data.^29^

**1‐[4‐Hydroxy‐5‐methoxy‐2‐(trifluoromethyl)phenyl]ethan‐1‐one (2c):** 33.2 mg (200 µmol, 1 equiv.) 1‐(4‐hydroxy‐3‐methoxyphenyl)ethan‐1‐one (**1c**), 48 h reaction time, purification via preparative reversed‐phase chromatography. Method: column oven 30 °C, flow rate 14 mL/min; 0.0–5.0 min MeCN/H_2_O = 3:97 (v/v), 5.0–30.0 min linear increase to MeCN/H_2_O = 60:40 (v/v), 30.0–31.0 min linear increase to MeCN/H_2_O = 100:0 (v/v), 31.0 min–36.0 min hold MeCN/H_2_O = 100:0 (v/v). GC–MS analysis of the crude product indicated a *major* (represents product **2c**) and two *minor* isomers with the relative ratio 17:2:1. Yield: 11.1 mg (47.4 µmol, 24 %) light‐yellow solid; *R*_f_ = 0.35 (cyclohexane/EtOAc 1:1); m.p. 108–111 °C; ^1^H NMR (300.13 MHz, CDCl_3_): *δ* = 7.23 (s, 1 H, CH_arom_), 6.94 (s, 1 H, CH_arom_), 5.91 (br. s, 1 H, OH), 3.97 (s, 3 H, OCH_3_), 2.56 ppm (s, 3 H, CH_3_); ^13^C NMR (75.47 MHz, CDCl_3_): *δ* = 201.2 (C_q_, C=O), 148.4 (C_q_), 147.1 (C_q_), 133.1 [C_q_, q, ^3^ *J*(C,F) = 2.0 Hz], 123.7 [q, ^1^ *J*(C,F) = 272.8 Hz, C_q_, CF_3_], 120.9 [C_q_, q, ^2^ *J*(C,F) = 33.1 Hz], 113.4 [q, ^3^ *J*(C,F) = 5.3 Hz, CH_arom_], 110.3 (CH_arom_), 56.4 (OCH_3_), 30.6 ppm (CH_3_); ^19^F NMR (282 MHz, CDCl_3_): *δ* = –56.92 ppm. MS (70 eV): *m/z* (%) = 234 [M^+^] (34), 220 (10), 219 (100), 191 (21), 176 (13), 148 (8), 120 (5), 43 (20); the spectra are in accordance with previously reported data.^29^

**Methyl 4,5‐dimethoxy‐2‐(trifluoromethyl)benzoate (2d):** 39.2 mg (200 µmol, 1 equiv.) methyl 3,4‐dimethoxybenzoate (**1d**), 48 h reaction time, purification via preparative reversed‐phase chromatography. Method: column oven 30 °C, flow rate 14 mL/min; 0.0–5.0 min MeCN/H_2_O = 3:97 (v/v), 5.0–30.0 min linear increase to MeCN/H_2_O = 70:30 (v/v), 30.0–31.0 min linear increase to MeCN/H_2_O = 100:0 (v/v), 31.0 min–36.0 min hold MeCN/H_2_O = 100:0 (v/v). GC–MS analysis of the crude product indicated a *major* (represents product **2d**) and a *minor* isomer with the relative ratio 10:1. Yield: 13.9 mg (52.6 µmol, 26 %) light yellow, viscous oil; *R*_f_ = 0.60 (cyclohexane/EtOAc, 1:1); ^1^H NMR (300.13 MHz, CDCl_3_): *δ* = 7.34 (s, 1 H, CH_arom_), 7.18 (s, 1 H, CH_arom_), 3.962 (s, 3 H, OCH_3_), 3.957 (s, 3 H, OCH_3_), 3.92 ppm (s, 3 H, OCH_3_); ^13^C NMR (125.69 MHz, CDCl_3_): *δ* = 166.9 (C_q_), 150.9 (C_q_), 150.8 (C_q_), 123.8 [C_q_, q, ^3^ *J*(C,F) = 2.0 Hz], 123.6 [q, ^1^ *J*(C,F) = 272.7 Hz, C_q_, CF_3_], 122.6 [C_q_, q, ^2^ *J*(C,F) = 33.3 Hz], 113.5 (CH_arom_), 109.7 [q, ^3^ *J*(C,F) = 5.8 Hz, CH_arom_], 56.4 (2 × CH_3_), 52.8 ppm (CH_3_); ^19^F NMR (282 MHz, CDCl_3_): *δ* = –58.47 ppm. IR (ATR): ν̃ = 2953, 2855, 1734, 1605, 1528, 1463, 1436, 1399, 1356, 1292, 1270, 1211, 1173, 1115, 1027, 987, 929, 876, 830, 791, 772, 744, 730, 694, 652, 567, 542 cm^–1^. MS (70 eV): *m/z* (%) = 264 [M^+^] (59), 234 (12), 233 (100), 205 (19), 193 (5), 190 (5), 189 (5), 187 (6), 161 (9), 147 (9), 119 (5), 69 (5). HR‐MS (HPLC‐TOF‐MS): *m/z* [M + NH_4_]^+^ calcd. for C_11_H_15_F_3_NO_4_^+^: 282.094769, found 282.094955.

**1‐[4‐Hydroxy‐3‐(trifluoromethyl)phenyl]ethan‐1‐one (2e):** 27.2 mg (200 µmol, 1 equiv.) 1‐(4‐hydroxyphenyl)ethan‐1‐one (**1e**), 48 h reaction time, purification via preparative reversed‐phase chromatography. Method: column oven 30 °C, flow rate 14 mL/min; 0.0–5.0 min MeCN/H_2_O = 3:97 (v/v), 5.0–30.0 min linear increase to MeCN/H_2_O = 55:45 (v/v), 30.0–31.0 min linear increase to MeCN/H_2_O = 100:0 (v/v), 31.0 min–36.0 min hold MeCN/H_2_O = 100:0 (v/v). Yield: 7.6 mg (37.2 µmol, 19 %) pale yellow solid; *R*_f_ = 0.40 (cyclohexane/EtOAc, 1:1); m.p. 171–172 °C; ^1^H NMR (300.13 MHz, [D_4_]MeOD): *δ* = 8.14 [d, ^4^ *J*(H,H) = 2.0 Hz, 1 H, CH_arom_], 8.06 [dd, ^3^ *J*(H,H) = 8.6, ^4^ *J*(H,H) = 2.1 Hz, 1 H, CH_arom_], 7.01 [d, ^3^ *J*(H,H) = 8.6 Hz, 1 H, CH_arom_], 5.48 (br. s, 1 H, OH), 2.56 ppm (s, 3 H, CH_3_); ^13^C NMR (75.47 MHz, [D_4_]MeOD): *δ* = 198.2 (C_q_, C=O), 161.7 [C_q_, q, ^3^ *J*(C,F) = 1.6 Hz], 135.2 (CH_arom_), 129.6 (C_q_), 128.9 [q, ^3^ *J*(C,F) = 5.2 Hz, CH_arom_], 125.0 [C_q_, q, ^1^ *J*(C,F) = 271.5 Hz], 118.1 [C_q_, q, ^2^ *J*(C,F) = 31.2 Hz], 117.7 (CH_arom_), 26.3 ppm (CH_3_); ^19^F NMR (282 MHz, [D_4_]MeOD): *δ* = –64.46 ppm. MS (70 eV): *m/z* (%) = 204 [M^+^] (33), 190 (6), 189 (71), 185 (9), 170 (8), 169 (100), 141 (12), 127 (5), 113 (25), 79 (5), 63 (18), 43 (17). The succession of signals in the NMR spectra in [D_4_]MeOD are in accordance with previously reported data in CDCl_3_.^29^

**4‐Hydroxy‐3‐(trifluoromethyl)benzonitrile (*major*‐2f)/4‐Hydroxy‐2‐(trifluoromethyl)benzonitrile (*minor*‐2f):** 23.8 mg (200 µmol, 1 equiv.) 4‐hydroxybenzonitrile (**1f**), 48 h reaction time, purification via preparative reversed‐phase chromatography. Method: column oven 30 °C, flow rate 14 mL/min; 0.0–5.0 min MeCN/H_2_O = 3:97 (v/v), 5.0–30.0 min linear increase to MeCN/H_2_O = 55:45 (v/v), 30.0–31.0 min linear increase to MeCN/H_2_O = 100:0 (v/v), 31.0 min–36.0 min hold MeCN/H_2_O = 100:0 (v/v). The product was obtained as an inseparable mixture of the *major* and *minor* regioisomer (ratio = 79:21, determined via ^1^H‐NMR integrals, 20 s delay). Yield: 10.8 mg (57.6 µmol, 29 %) pale yellow solid; *R*_f_ = 0.41 (cyclohexane/EtOAc, 1:1); m.p. 154–158 °C; ^1^H NMR (300.13 MHz, [D_4_]MeOD): *δ* = 7.89 [d, ^4^ *J*(H,H) = 2.0 Hz, 1 H, C‐2_major_], 7.81–7.73 (m, 1.28 H, C‐6_major_, C‐6_minor_), 7.22 [d, ^4^ *J*(H,H) = 2.4 Hz, 0.27 H, C‐3_minor_], 7.14–7.05 ppm (m, 1.28 H, C‐5_major_, C‐5_minor_); ^13^C NMR (75.47 MHz, CDCl_3_): *δ* = 159.8 (C‐4_minor_), 157.7 [q, ^3^ *J*(C,F) = 1.7 Hz, C‐4_major_], 137.4 [q, ^4^ *J*(C,F) = 0.6 Hz,C‐6_major_], 137.0 (C‐6_minor_), 131.9 [q, ^3^ *J*(C,F) = 5.0 Hz, C‐2_major_], 122.9 [q, ^1^ *J*(C,F) = 273.0 Hz, CF_3,major_], 119.0 (C‐5_minor_), 118.9 (C‐5_major_), 118.1 [q, ^2^ *J*(C,F) = 32.0 Hz, C‐3_major_], 117.8 (CN_major_), 115.9 (CN_minor_), 114.8 [q, ^3^ *J*(C,F) = 4.7 Hz, C‐3_minor_], 104.3 (C‐1_major_), 101.2 ppm (C‐1_minor_); ^13^C NMR (75.47 MHz, [D_6_]acetone): *δ* = 161.7 (C‐4_minor_), 159.6 [q, ^3^ *J*(C,F) = 1.7 Hz, C‐4_major_], 137.6 (C‐6_major_), 137.2 (C‐6_minor_), 131.6 [q, ^3^ *J*(C,F) = 5.4 Hz, C‐2_major_], 123.1 [q, ^1^ *J*(C,F) = 271.8 Hz, CF_3,major_], 119.4 (C‐5_minor_), 118.2 (C‐5_major_), 117.8 (CN_major_), 177.7 [q, ^2^ *J*(C,F) = 31.6 Hz, C‐3_major_], 114.3 [q, ^3^ *J*(C,F) = 5.1 Hz, C‐3_minor_], 102.7 ppm (C‐1_major_). The remaining quaternary C‐atoms of the *minor*‐isomer were not detected due to low intensity. ^19^F NMR (282 MHz, [D_4_]MeOD): *δ* = –63.89 (*minor*), –64.82 ppm (*major*). MS (70 eV): *m/z* (%) = 187 [M^+^] (54), 168 (19), 167 (83), 140 (8), 139 (100), 113 (5), 112 (9), 88 (17), 75 (14), 69 (7), 64 (8), 63 (12), 62 (8), 44 (5). The spectra are in accordance with previously reported data.^29^

## Supporting information

Supporting Information
