# Supplementary material for: Cu and Hydroquinone for the Trifluoromethylation of Unprotected Phenols
Source: European J Org Chem. 2018 Oct 8;2019(4):682–90. doi: 10.1002/ejoc.201801111 (PMC6391967; doi:10.1002/ejoc.201801111)
Supplement: Supplementary file 1 — Supporting Information [file EJOC-2019-682-s001.pdf]

Supporting Information

Cu and Hydroquinone for the Trifluoromethylation of Unprotected Phenols

Jakob Pletz, Christoph Koeberl, Michael Fuchs, Oliver Steiner, Walter Goessler, and Wolfgang Kroutil*

ejoc201801111-sup-0001-SupMat.pdf

Table of Contents

SI-1. Experimental Procedures 3

SI-2. Experimental Spectra 19

SI-3. References 30

SI-1. Experimental Procedures

Test reaction: Laccase-catalyzed trifluoromethylation

Eppendorf vials (1.5 mL) were charged with a freshly prepared DMSO stock solution (63 µL) of acetosyringone (**1a**) (2.5 mg, 12.5 µmol, 1 eq.), $Zn(SO_2CF_3)_2 \cdot 2\ H_2O$ (8.3 mg, 25 µmol, 2 eq.) and hydroquinone (0.7 mg, 6.25 µmol, 0.5 eq.). Laccase (1.25 mg, *A. bisporus*, >4 U mg^{-1}, final concentration: 5 mg mL^{-1}) dissolved in NaOAc buffer (174 µL, 250 mM, pH 5.5) was added. The vial was sealed and shaken by hand, an aliquot was withdrawn for GC-FID analysis and 70% aqueous TBHP solution (14.3 µL, 100 µmol, 8 eq.) was added in one portion at 22 °C. The vials were sealed and shaken in an orbital shaker (30 °C, 900 rpm, horizontal position) for 24 h. An aliquot of the reaction mixture (25.0 µL) was transferred into a separate Eppendorf vial (1.5 mL), mixed with standard solution (200 µL, 6.25 µM 4'-methoxyacetophenone in EtOAc), the vial was vortexed (60 s) and centrifuged. The phases were separated and the organic layer was dried over Na_2SO_4 and analyzed by GC-FID.

Determination of the copper concentration in the native & desalted laccase solution via ICP-MS

Laccase from *A. bisporus* (7.2 mg) was dissolved in NaOAc buffer (500 µL, 250 mM, pH 5.5) and was desalted using a PD MiniTrap G-25 column (GE Healthcare) according to the manufacturer's manual giving a desalted laccase solution (1000 mL, 7.2 mg mL^{-1}). The total copper concentrations of the desalted (7.2 mg mL^{-1}) and not-desalted laccase solution (in NaOAc buffer (250 mM, pH 5.5), 5 mg mL^{-1}) were determined by ICPMS (Table SI-1). The desalted laccase solution was used for the trifluoromethylation reactions straight after the desalting procedure. A concentration of 7.2 mg L^{-1} was prepared and measured via ICPMS, because the dilution step in the subsequent trifluoromethylation reaction gave the needed concentration of 5 mg mL^{-1}.

Table SI-1. Determination of copper concentration in laccase and desalted laccase solution via ICPMS.

Entry	Sample[a,b]	c (Cu) [mg L^{-1}]	c (Cu) [µmol L^{-1}]	c (Cu) [ng mg^{-1} laccase]
1	Laccase (5 mg mL^{-1})	19.2	302	3840
2	desalted laccase (7.2 mg mL^{-1})	1.9	29.8	264

[a] samples were dissolved in NaOAc buffer (250 mM, pH 5.5), [b] laccase from *A. bisporus* was used.

Test reactions: Trifluoromethylation with desalted laccase (without CuI)

Eppendorf vials (1.5 mL) were charged with a freshly prepared DMSO stock solution (63 µL) of acetosyringone (**1a**) (2.5 mg, 12.5 µmol, 1 eq.), $Zn(SO_2CF_3)_2 \cdot 2\ H_2O$ (8.3 mg, 25 µmol, 2 eq.) and hydroquinone (0.7 mg, 6.25 µmol, 0.5 eq.). Desalted laccase solution (174 µL, final concentration: 5 mg mL^{-1}) dissolved in NaOAc buffer (174 µL, 250 mM, pH 5.5) was added. The vial was sealed and shaken by hand, an aliquot was taken for GC-FID analysis and 70% aqueous TBHP solution (14.3 µL, 100 µmol, 8 eq.) was added in one portion at 22 °C. The vials were sealed and shaken in an orbital shaker (30 °C, 900 rpm, horizontal position) for 24 h. An aliquot of the reaction mixture (25.0 µL) was transferred into a separate Eppendorf vial (1.5 mL), mixed with standard solution (200 µL, 6.25 µM 4'-methoxyacetophenone in EtOAc), the vial was vortexed (60 s) and centrifuged. The phases were separated and the organic layer was dried over Na_2SO_4 and analyzed by GC-FID.

Test reactions: Trifluoromethylation with desalted laccase (with CuI)

Eppendorf vials (1.5 mL) were charged with a freshly prepared DMSO stock solution (63 µL) of acetosyringone (**1a**) (2.5 mg, 12.5 µmol, 1 eq.), $Zn(SO_2CF_3)_2 \cdot 2\ H_2O$ (8.3 mg, 25 µmol, 2 eq.) and hydroquinone (0.7 mg, 6.25 µmol, 0.5 eq.). Desalted laccase solution (174 µL, 7.2 mg mL^{-1}, final concentration: 5 mg mL^{-1}) in NaOAc buffer (250 mM, pH 5.5) was added followed by a DMSO stock solution of CuI (10 µL, 4.0 mM, 50 nmol, 0.004 eq.). The vial was sealed and shaken by hand, an aliquot was taken for GC-FID analysis and 70% aqueous TBHP solution (14.3 µL, 100 µmol, 8 eq.) was added in one portion at 22 °C. The vials were sealed and shaken in an orbital shaker (30 °C, 900 rpm, horizontal position) for 24 h. An aliquot of the reaction mixture (25.0 µL) was transferred into a separate Eppendorf vial (1.5 mL), mixed with standard solution (200 µL, 6.25 µM 4'-methoxyacetophenone in EtOAc), the vial was vortexed (60 s) and centrifuged. The phases were separated and the organic layer was dried over Na_2SO_4 and analyzed by GC-FID.

Photometric Enzymatic Activity Assay

The enzymatic activities of the laccase originating from *A. bisporus* (0.5 mg mL^{-1}), the desalted laccase from A. bisporus (0.5 mg mL^{-1}) as well as the mixture of the desalted laccase from *A. bisporus* (0.5 mg mL^{-1}) and CuI (200 µM) was determined spectrophotometrically (λ = 469 nm) for ten minutes in triplicate at room temperature according to a literature procedure.[1] A SpectraMax M2 plate reader from Molecular Devices was used. 2,6-Dimethoxyphenol (DMP, 0.83 mg mL^{-1}) was employed as model substrate with deionized water (pH 6.0) as reaction medium. The amount of DMSO was 25 vol% to ensure authentic reaction conditions. We performed a photometric enzymatic activity assay with 2,6-dimethoxyphenol using a laccase-, desalted laccase- and desalted laccase solution supplemented with 0.004 eq. Cu(I) iodide (Table SI-2).

Table SI-2. Photometric enzymatic activity assay with 2,6-dimethoxyphenol.

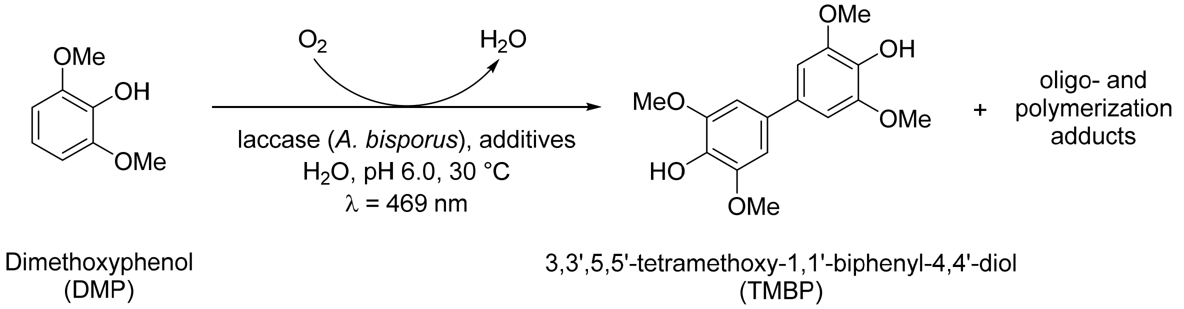

Entry	Catalyst	Additive	k_{max} (mean) [mAU min^{-1}]	$k_{m,c}$ (M min^{-1})[d]	turnover frequency (s^{-1})
1	laccase[a]	-	556	11.2	24276
2	desalted laccase[b]	-	457	9.20	19944
3	desalted laccase[b]	CuI[c]	588	11.9	25684

[a] 5 g L^{-1} laccase (*A. bisporus*). [b] 5 g L^{-1} desalted laccase (*A. bisporus*). [c] 0.004 eq. CuI. [d] ε_{469}(DMP) = 49.600 M^{-1} cm^{-1}.[2]

Test reactions: CuI-catalyzed trifluoromethylation (without hydroquinone)

An Eppendorf vial (1.5 mL) was charged with a freshly prepared DMSO stock solution (63 µL) of acetosyringone (2.5 mg, 12.5 µmol, 1 eq.) and $Zn(SO_2CF_3)_2 \cdot 2\ H_2O$ (8.3 mg, 25 µmol, 2 eq.), followed by a stock solution of CuI (10 µL, 4.0 mM, 50 nmol, 0.004 eq.). NaOAc buffer (174 µL, 250 mM, pH 5.5) was added, the vials were sealed and mixed thoroughly (vortex), an aliquot was taken for GC-FID analysis and 70% aqueous TBHP solution (14.3 µL, 100 µmol, 8 eq.) was added in one portion at 22 °C. The vial was sealed and shaken in an orbital shaker (30 °C, 900 rpm, horizontal position) for 24 h. An aliquot of the reaction mixture (25.0 µL) was transferred into a separate Eppendorf vial (1.5 mL) with standard solution (200 µL, 6.25 µM

4'-methoxyacetophenone in EtOAc), the vial was vortexed (60 s) and centrifuged. The phases were separated and the organic layer was dried over Na_2SO_4 and analyzed by GC-FID.

Test reactions: Cu-catalyzed trifluoromethylation (with hydroquinone)

An Eppendorf vial (1.5 mL) was charged with a freshly prepared DMSO stock solution (63 µL) of acetosyringone (2.5 mg, 12.5 µmol, 1 eq.), $Zn(SO_2CF_3)_2 \cdot 2\ H_2O$ (8.3 mg, 25 µmol, 2 eq.) and hydroquinone (0.7 mg, 6.25 µmol, 0.5 eq.) followed by a stock solution of CuI (10 µL, 4.0 mM, 50 nmol, 0.004 eq.). NaOAc buffer (174 µL, 250 mM, pH 5.5) was added, the vial was sealed and mixed thoroughly (vortex), an aliquot was taken for GC-FID analysis and 70% aqueous TBHP solution (14.3 µL, 100 µmol, 8 eq.) was added in one portion at 22 °C. The vial was sealed and shaken in an orbital shaker (30 °C, 900 rpm, horizontal position) for 24 h. An aliquot of the reaction mixture (25.0 µL) was transferred into a separate Eppendorf vial (1.5 mL) with standard solution (200 µL, 6.25 µM 4'-methoxyacetophenone in EtOAc), the vial was vortexed (60 s) and centrifuged. The phases were separated and the organic layer was dried over Na_2SO_4 and analyzed by GC-FID.

Cu-catalyzed trifluoromethylation at varied amounts of hydroquinone

Eppendorf vials (1.5 mL) were charged with a freshly prepared DMSO stock solution (63 µL) of acetosyringone (2.5 mg, 12.5 µmol, 1 eq.) and $Zn(SO_2CF_3)_2 \cdot 2\ H_2O$ (8.3 mg, 25 µmol, 2 eq.), followed by a stock solution of CuI (10 µL, 4.0 mM, 50 nmol, 0.004 eq.). The respective amount of hydroquinone was added as a DMSO stock solution (10 µL) followed by NaOAc buffer (174 µL, 250 mM, pH 5.5). The vials were sealed and mixed thoroughly (vortex), an aliquot was taken for GC-FID analysis and 70% aqueous TBHP solution (14.3 µL, 100 µmol, 8 eq.) was added in one portion at 22 °C. The vials were sealed and shaken in an orbital shaker (30 °C, 900 rpm, horizontal position) for 24 h. An aliquot of the reaction mixture (25.0 µL) was transferred into a separate Eppendorf vial (1.5 mL) with standard solution (200 µL, 6.25 µM 4'-methoxyacetophenone in EtOAc), the vial was vortexed (60 s) and centrifuged. The phases were separated and the organic layer was dried over Na_2SO_4 and analyzed by GC-FID.

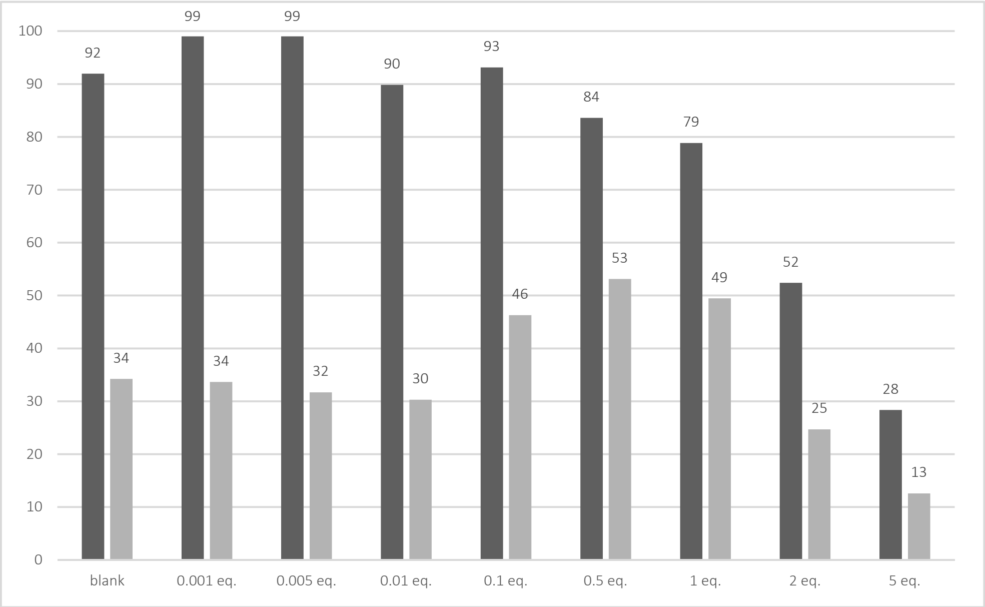

Figure SI-1. CuI-catalyzed trifluoromethylation at varied amounts of HQ. Conditions: 1 eq. acetosyringone (**1a**, 12.5 µmol), 2 eq. $Zn(SO_2CF_3)_2$, 0.004 eq. CuI, 8 eq. TBHP, 25 vol% DMSO, NaOAc buffer (250 mM, pH 5.5), 30 °C, 900 rpm, 24 h. Dark grey bars: conversion of **1a**; light grey bars: yield **2a**.

Laccase-catalyzed trifluoromethylation at varied amounts of hydroquinone

Eppendorf vials (1.5 mL) were charged with a freshly prepared DMSO stock solution (63 µL) of acetosyringone (2.5 mg, 12.5 µmol, 1 eq.) and $Zn(SO_2CF_3)_2 \cdot 2\ H_2O$ (8.3 mg, 25 µmol, 2 eq.) followed the respective amount of hydroquinone as a DMSO stock solution (10 µL). Laccase (1.25 mg, *A. bisporus*, >4 U mg^{-1}, 5 mg mL^{-1}) dissolved in NaOAc buffer (174 µL, 250 mM, pH 5.5) was added. The vials were sealed, shaken by hand, an aliquot was taken for GC-FID analysis and 70% aqueous TBHP solution (14.3 µL, 100 µmol, 8 eq.) was added in one portion at 22 °C. The vials were sealed and shaken in an orbital shaker (30 °C, 900 rpm, horizontal position) for 24 h. An aliquot of the reaction mixture (25.0 µL) was transferred into a separate Eppendorf vial (1.5 mL) with standard solution (200 µL, 6.25 µM 4'-methoxyacetophenone in EtOAc), the vial was vortexed (60 s) and centrifuged. The phases were separated and the organic layer was dried over Na_2SO_4 and analyzed by GC-FID.

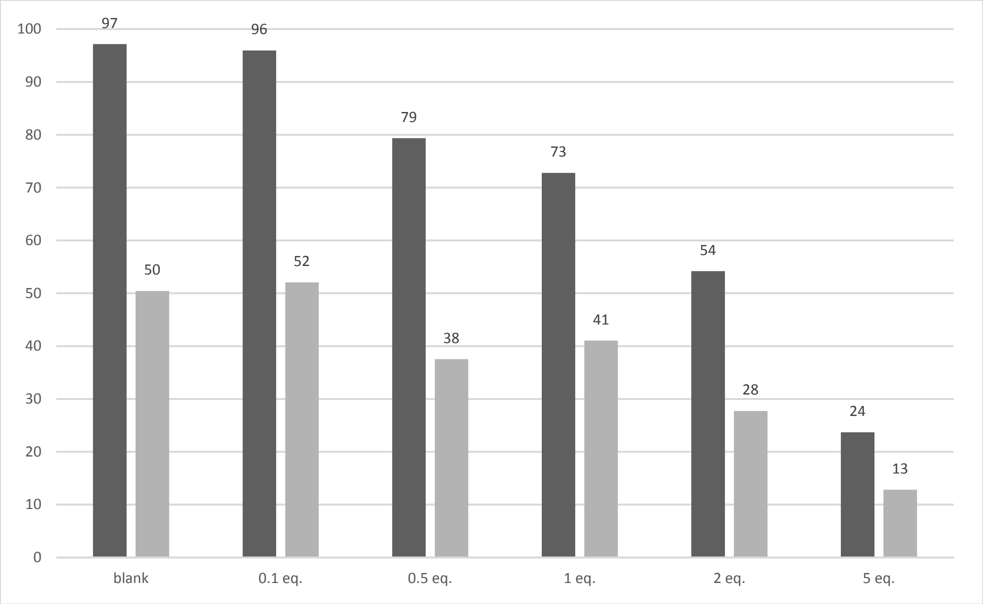

Figure SI-2. Laccase-catalyzed trifluoromethylation at varied amounts of HQ. Conditions: 5 mg mL^{-1} laccase from *A. bisporus*, 1 eq. acetosyringone (**1a**, 12.5 µmol), 2 eq. $Zn(SO_2CF_3)_2$, 8 eq. TBHP, 25 vol% DMSO, NaOAc buffer (250 mM, pH 5.5), 30 °C, 900 rpm, 24 h. Dark grey bars: conversion of **1a**; light grey bars: yield **2a**.

HQ-mediated trifluoromethylation at varied amounts of CuI

Eppendorf vials (1.5 mL) were charged with a freshly prepared DMSO stock solution (63 µL) of acetosyringone (2.5 mg, 12.5 µmol, 1 eq.), $Zn(SO_2CF_3)_2 \cdot 2\ H_2O$ (8.3 mg, 25 µmol, 2 eq.) and hydroquinone (0.7 mg, 6.25 µmol, 0.5 eq.). A DMSO stock solution (10 mL) of the respective amount of CuI was added followed by NaOAc buffer (174 µL, 250 mM, pH 5.5). The vials were sealed and mixed thoroughly (vortex), an aliquot was taken for GC-FID analysis and 70% aqueous TBHP solution (14.3 µL, 100 µmol, 8 eq.) was added in one portion at 22 °C. The vials were sealed and shaken in an orbital shaker (30 °C, 900 rpm, horizontal position) for 24 h. An aliquot of the reaction mixture (25.0 µL) was transferred into a separate Eppendorf vial (1.5 mL) with standard solution (200 µL, 6.25 µM 4'-methoxyacetophenone in EtOAc), the vial was vortexed (60 s) and centrifuged. The phases were separated and the organic layer was dried over Na_2SO_4 and analyzed by GC-FID.

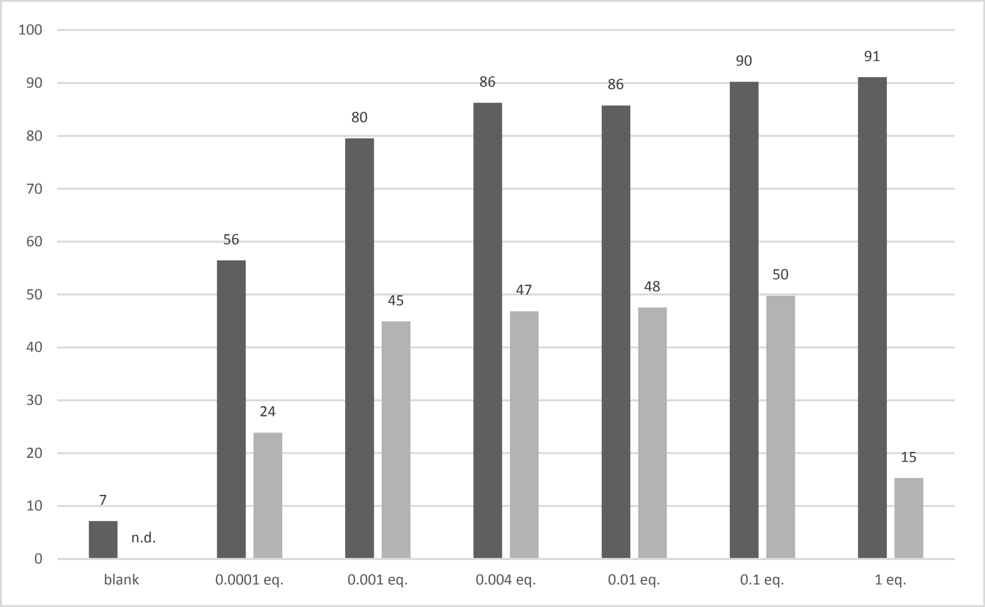

Figure SI-3. HQ-mediated trifluoromethylation at varied amounts of CuI. Conditions: 1 eq. acetosyringone (**1a**, 12.5 µmol), 0.5 eq. HQ, 2 eq. $Zn(SO_2CF_3)_2$, 8 eq. TBHP, 25 vol% DMSO, NaOAc buffer (250 mM, pH 5.5), 30 °C, 900 rpm, 24 h. Dark grey bars: conversion of **1a**; light grey bars: yield **2a**.

Trifluoromethylation without HQ at varied amounts of CuI

Eppendorf vials (1.5 mL) were charged with a freshly prepared DMSO stock solution (63 µL) of acetosyringone (2.5 mg, 12.5 µmol, 1 eq.) and $Zn(SO_2CF_3)_2 \cdot 2\ H_2O$ (8.3 mg, 25 µmol, 2 eq.). A DMSO stock solution (10 mL) of the respective amount of CuI was added followed by NaOAc buffer (174 µL, 250 mM, pH 5.5). The vials were sealed and mixed thoroughly (vortex), an aliquot was taken for GC-FID analysis and 70% aqueous TBHP solution (14.3 µL, 100 µmol, 8 eq.) was added in one portion at 22 °C. The vials were sealed and shaken in an orbital shaker (30 °C, 900 rpm, horizontal position) for 24 h. An aliquot of the reaction mixture (25.0 µL) was transferred into a separate Eppendorf vial (1.5 mL) with standard solution (200 µL, 6.25 µM4'-methoxyacetophenone in EtOAc), the vial was vortexed (60 s) and centrifuged. The phases were separated and the organic layer was dried over Na_2SO_4 and analyzed by GC-FID.

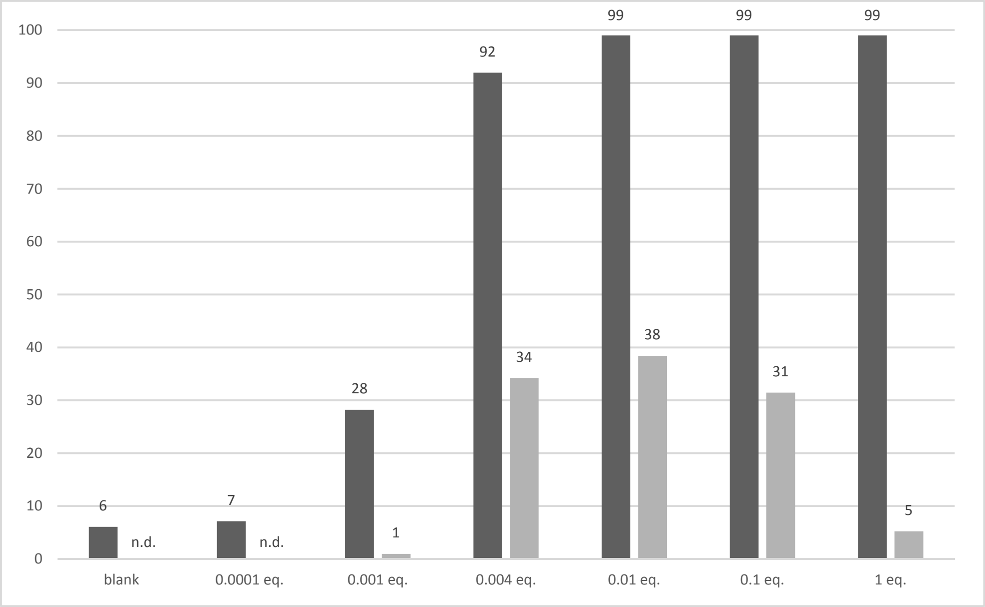

Figure SI-4. Trifluoromethylation without HQ at varied amounts of CuI. Conditions: 1 eq. acetosyringone (**1a**, 12.5 µmol), 2 eq. $Zn(SO_2CF_3)_2$, 8 eq. TBHP, 25 vol% DMSO, NaOAc buffer (250 mM, pH 5.5), 30 °C, 900 rpm, 24 h. Dark grey bars: conversion of **1a**; light grey bars: yield **2a**.

Effect of different copper salts on the Cu-catalyzed HQ-mediated trifluoromethylation

Eppendorf vials (1.5 mL) were charged with a freshly prepared DMSO stock solution (63 µL) of acetosyringone (2.5 mg, 12.5 µmol, 1 eq.), $Zn(SO_2CF_3)_2 \cdot 2\,H_2O$ (8.3 mg, 25 µmol, 2 eq.) and hydroquinone (0.7 mg, 6.25 µmol, 0.5 eq.). An aqueous stock solution (except CuI: DMSO) of the respective amount of copper salt (10 µL, 50.0 nM, 0.004 eq.) was added followed by NaOAc buffer (174 µL, 250 mM, pH 5.5). The vials were sealed and mixed thoroughly (vortex), an aliquot was taken for GC-FID analysis and 70% aqueous TBHP solution (14.3 µL, 100 µmol, 8 eq.) was added in one portion at 22 °C. The vials were sealed and shaken in an orbital shaker (30 °C, 900 rpm, horizontal position) for 24 h. An aliquot of the reaction mixture (25.0 µL) was transferred into a separate Eppendorf vial (1.5 mL) with standard solution (200 µL, 6.25 µM 4'-methoxyacetophenone in EtOAc), the vial was vortexed (60 s) and centrifuged. The phases were separated and the organic layer was dried over Na_2SO_4 and analyzed by GC-FID.

Table SI-3. Effect of different copper salts on the HQ-mediated Cu-catalyzed trifluoromethylation.[a]

Entry	Catalyst [0.004 eq.]	Conversion [%][b]	Yield **2a** [%][b]
1	$Cu(OAc)_2$	90	40
2	$CuSO_4$	91	42
3	$CuBr_2$	91	43
4	$CuCl_2$	90	43
5	CuI	88	45
6	$Cu(NO_3)_2$	88	45

[a] conditions: 1 eq. acetosyringone (**1a**, 12.5 µmol), 2 eq. $Zn(SO_2CF_3)_2$, 0.5 eq. HQ, 8 eq. TBHP, 25 vol% DMSO, NaOAc buffer (250 mM, pH 5.5), 30 °C, 900 rpm, 24 h. [b] determined via GC-FID using 4'-methoxyacetophenone as internal standard.

HQ-mediated Cu-catalyzed trifluoromethylation at varied amounts of TBHP

Eppendorf vials (1.5 mL) were charged with a freshly prepared DMSO stock solution (63 µL) of acetosyringone (2.5 mg, 12.5 µmol, 1 eq.), $Zn(SO_2CF_3)_2{\cdot}2\ H_2O$ (8.3 mg, 25 µmol, 2 eq.) and hydroquinone (0.7 mg, 6.25 µmol, 0.5 eq.). A stock solution of CuI (10 µL, 4.0 mM, 50 nmol, 0.004 eq.) was added followed by NaOAc buffer (174 µL, 250 mM, pH 5.5). The vials were sealed and mixed thoroughly (vortex), an aliquot was taken for GC-FID analysis and the respective amount of 70% aqueous TBHP solution was added in one portion at 22 °C. The vials were sealed and shaken in an orbital shaker (30 °C, 900 rpm, horizontal position) for 24 h. An aliquot of the reaction mixture (25.0 µL) was transferred into a separate Eppendorf vial (1.5 mL) with standard solution (200 µL, 6.25 µM 4'-methoxyacetophenone in EtOAc), the vial was vortexed (60 s) and centrifuged. The phases were separated and the organic layer was dried over Na_2SO_4 and analyzed by GC-FID.

Table SI-4. HQ-mediated Cu-catalyzed trifluoromethylation at varied amounts of TBHP.[a]

Entry	TBHP [eq.]	Conversion [%][b]	Yield **2a** [%][b]
1	0.5	14	6
2	1	27	13
3	2	58	32
4	4	91	50
5	6	94	51
6	8	93	49
7	10	91	47

[a] conditions: 1 eq. acetosyringone (**1a**, 12.5 µmol), 2 eq. $Zn(SO_2CF_3)_2$, 0.5 eq. HQ, 0.004 eq. CuI, 25 vol% DMSO, NaOAc buffer (250 mM, pH 5.5), 30 °C, 900 rpm, 24 h. [b] determined via GC-FID using 4'-methoxyacetophenone as internal standard.

HQ-mediated Cu-catalyzed trifluoromethylation at varied amounts of $Zn(SO_2CF_3)_2$

Eppendorf vials (1.5 mL) were charged with a freshly prepared DMSO stock solution (63 µL) of acetosyringone (2.5 mg, 12.5 µmol, 1 eq.) and hydroquinone (0.7 mg, 6.25 µmol, 0.5 eq.). A stock solution of CuI (10 µL, 4.0 mM, 50 nmol, 0.004 eq.) was added followed by the respective amount of $Zn(SO_2CF_3)_2 \cdot 2\ H_2O$ and NaOAc buffer (174 µL, 250 mM, pH 5.5). The vials were sealed and mixed thoroughly (vortex), an aliquot was taken for GC-FID analysis and the respective amount of 70% aqueous TBHP solution was added in one portion at 22 °C. The vials were sealed and shaken in an orbital shaker (30 °C, 900 rpm, horizontal position) for 24 h. An aliquot of the reaction mixture (25.0 µL) was transferred into a separate Eppendorf vial (1.5 mL) with standard solution (200 µL, 6.25 µM 4'-methoxyacetophenone in EtOAc), the vial was vortexed (60 s) and centrifuged. The phases were separated and the organic layer was dried over Na_2SO_4 and analyzed by GC-FID.

Table SI-5. HQ-mediated Cu-catalyzed trifluoromethylation at varied amounts of $Zn(SO_2CF_3)_2$.[a]

Entry	$Zn(SO_2CF_3)_2$ [eq.]	Conversion [%][b]	Yield **2a** [%][b]
1	0.5	31	12
2	1	56	26
3	1.5	72	34
4	2	89	57
5	2.5	99	55
6	3	99	47
7	4	98	38

[a] conditions: 1 eq. acetosyringone (**1a**, 12.5 µmol), 0.004 eq. CuI, 0.5 eq. HQ, 8 eq. TBHP, 25 vol% DMSO, NaOAc buffer (250 mM, pH 5.5), 30 °C, 900 rpm, 24 h. [b] determined via GC-FID using 4'-methoxyacetophenone as internal standard.

HQ-mediated Cu-catalyzed trifluoromethylation at varied pH

Eppendorf vials (1.5 mL) were charged with a freshly prepared DMSO stock solution (63 µL) of acetosyringone (2.5 mg, 12.5 µmol, 1 eq.), $Zn(SO_2CF_3)_2 \cdot 2\ H_2O$ (8.3 mg, 25 µmol, 2 eq.) and hydroquinone (0.7 mg, 6.25 µmol, 0.5 eq.). A stock solution of CuI (10 µL, 4.0 mM, 50 nmol, 0.004 eq.) was added followed by the respective buffer (174 µL, 100 mM). The vials were sealed and mixed thoroughly (vortex), an aliquot was taken for GC-FID analysis and the respective amount of 70% aqueous TBHP solution was added in one portion at 22 °C. The vials were sealed and shaken in an orbital shaker (30 °C, 900 rpm, horizontal position) for 24 h. An aliquot of the reaction mixture (25.0 µL) was transferred into a separate Eppendorf vial (1.5 mL) with standard solution (200 µL, 6.25 µM4'-methoxyacetophenone in EtOAc), the vial was vortexed (60 s) and centrifuged. The phases were separated and the organic layer was dried over Na_2SO_4 and analyzed by GC-FID. Following buffers (100 mM) were used: pH 5.0 (citric acid - sodium dihydrogenphosphate), pH 6.0 (sodium phosphate), pH 7.0 (sodium phosphate), pH 8.0 (sodium phosphate), pH 9.0 (Tris-HCl), pH 10.0 (glycine-NaOH), pH 11.0 (glycine-NaOH).

Table SI-6. HQ-mediated Cu-catalyzed trifluoromethylation at varied pH.[a]

Entry	pH value	Conversion [%][b]	Yield **2a** [%][b]
1	5	89	48
2	6	91	47
3	7	93	49
4	8	81	44
5	9	53	21
6	10	28	11
7	11	19	9

[a] conditions: buffer (100 mM), 1 eq. acetosyringone (**1a**, 12.5 µmol), 2 eq. $Zn(SO_2CF_3)_2$, 0.004 eq. CuI, 0.5 eq. HQ, 8 eq. TBHP, 25 vol% DMSO, 30 °C, 900 rpm, 24 h. [b] determined via GC-FID using 4'-methoxyacetophenone as internal standard.

Effect of different co-solvents on the Cu-catalyzed HQ-mediated trifluoromethylation

Eppendorf vials (1.5 mL) were charged with a MeCN stock solution of CuI (10 µL, 4.0 mM, 50 nmol, 0.004 eq.) and the solvent was removed in vacuum. Acetosyringone (2.5 mg, 12.5 µmol, 1 eq.), $Zn(SO_2CF_3)_2{\cdot}2\,H_2O$ (8.3 mg, 25 µmol, 2 eq.) and hydroquinone (0.7 mg, 6.25 µmol, 0.5 eq.) were added followed by the respective co-solvent (63 µL) and NaOAc buffer (174 µL, 250 mM, pH 5.5). The vials were sealed and mixed thoroughly (vortex), an aliquot was taken for GC-FID analysis and the respective amount of 70% aqueous TBHP solution was added in one portion at 22 °C. The vials were sealed and shaken in an orbital shaker (30 °C, 900 rpm, horizontal position) for 24 h. An aliquot of the reaction mixture (25.0 µL) was transferred into a separate Eppendorf vial (1.5 mL) with standard solution (200 µL, 6.25 µM4'-methoxyacetophenone in EtOAc), the vial was vortexed (60 s) and centrifuged. The phases were separated and the organic layer was dried over Na_2SO_4 and analyzed by GC-FID.

Table SI-7. Effect of co-solvent on the HQ-mediated Cu-catalyzed trifluoromethylation.[a]

Entry	Co-solvent (25 vol%)	Conversion [%][b]	Yield **2a** [%][b]
1	2-propanol	36	14
2	MeCN	69	36
3	acetone	76	39
4	DMSO	87	55

[a] conditions: 1 eq. acetosyringone (**1a**, 12.5 µmol), 2 eq. $Zn(SO_2CF_3)_2$, 0.004 eq. CuI, 0.5 eq. HQ, 8 eq. TBHP, 25 vol% DMSO, NaOAc buffer (250 mM, pH 5.5), 30 °C, 900 rpm, 24 h. [b] determined via GC-FID using 4'-methoxyacetophenone as internal standard.

Influence of TEMPO on the HQ-mediated Cu-catalyzed trifluoromethylation reaction

Eppendorf vials (1.5 mL) were charged with a freshly prepared DMSO stock solution (63 µL) of acetosyringone (2.5 mg, 12.5 µmol, 1 eq.) and $Zn(SO_2CF_3)_2{\cdot}2\,H_2O$ (8.3 mg, 25 µmol, 2 eq.) followed by the additive hydroquinone (0.7 mg, 6.25 µmol, 0.5 eq.) and/or quencher TEMPO (2.0 mg, 12.5 µmol, 1 eq.). A stock solution of CuI (10 µL, 4.0 mM, 50 nmol, 0.004 eq.) was added followed by NaOAc buffer (174 µL, 250 mM, pH 5.5). The vials were sealed and mixed thoroughly (vortex), an aliquot was taken for GC-FID analysis and the respective amount of 70% aqueous TBHP solution was added in one portion at 22 °C. The vials were sealed and shaken in an orbital shaker (30 °C, 900 rpm, horizontal position) for 24 h. An aliquot of the

reaction mixture (25.0 µL) was transferred into a separate Eppendorf vial (1.5 mL) with standard solution (200 µL, 6.25 µM 4'-methoxyacetophenone in EtOAc), the vial was vortexed (60 s) and centrifuged. The phases were separated and the organic layer was dried over Na_2SO_4 and analyzed by GC-FID.

Effect of hydroquinone and benzoquinone on the Cu-catalyzed trifluoromethylation

Eppendorf vials (1.5 mL) were charged with a freshly prepared DMSO stock solution (63 µL) of acetosyringone (2.5 mg, 12.5 µmol, 1 eq.), $Zn(SO_2CF_3)_2 \cdot 2\ H_2O$ (8.3 mg, 25 µmol, 2 eq.) and hydroquinone (0.7 mg, 6.25 µmol, 0.5 eq.) or benzoquinone (0.7 mg, 6.25 µmol, 0.5 eq.). A stock solution of CuI (10 µL, 4.0 mM, 50 nmol, 0.004 eq.) was added followed by NaOAc buffer (174 µL, 250 mM, pH 5.5). The vials were sealed and mixed thoroughly (vortex), an aliquot was taken for GC-FID analysis and 70% aqueous TBHP solution (14.3 µL, 100 µmol, 8 eq.) was added in one portion at 22 °C. The vials were sealed and shaken in an orbital shaker (30 °C, 900 rpm, horizontal position) for 24 h. An aliquot of the reaction mixture (25.0 µL) was transferred into a separate Eppendorf vial (1.5 mL) with standard solution (200 µL, 6.25 µM 4'-methoxyacetophenone in EtOAc), the vial was vortexed (60 s) and centrifuged. The phases were separated and the organic layer was dried over Na_2SO_4 and analyzed by GC-FID.

Effect of hydroquinone and benzoquinone on the laccase-catalyzed trifluoromethylation

Eppendorf vials (1.5 mL) were charged with a freshly prepared DMSO stock solution (63 µL) of acetosyringone (2.5 mg, 12.5 µmol, 1 eq.), $Zn(SO_2CF_3)_2 \cdot 2\ H_2O$ (8.3 mg, 25 µmol, 2 eq.) and hydroquinone (0.7 mg, 6.25 µmol, 0.5 eq.) or benzoquinone (0.7 mg, 6.25 µmol, 0.5 eq.). Laccase (1.25 mg, *A. bisporus*, >4 U mg^{-1}, 5 mg mL^{-1}) dissolved in NaOAc buffer (174 µL, 250 mM, pH 5.5) was added. The vials were sealed, shaken by hand, an aliquot was taken for GC-FID analysis and 70% aqueous TBHP solution (14.3 µL, 100 µmol, 8 eq.) was added in one portion at 22 °C. The vials were sealed and shaken in an orbital shaker (30 °C, 900 rpm, horizontal position) for 24 h. An aliquot of the reaction mixture (25.0 µL) was transferred into a separate Eppendorf vial (1.5 mL) with standard solution (200 µL, 6.25 µM 4'-methoxyacetophenone in EtOAc), the vial was vortexed (60 s) and centrifuged. The phases were separated and the organic layer was dried over Na_2SO_4 and analyzed by GC-FID.

Short-time kinetics of the Cu-catalyzed trifluoromethylation at varied amounts of HQ

Eppendorf vials (1.5 mL) were charged with a freshly prepared DMSO stock solution (106 µL) of acetosyringone (5.0 mg, 25.0 µmol, 1 eq.) and $Zn(SO_2CF_3)_2 \cdot 2\ H_2O$ (16.6 mg, 50 µmol,

2 eq.), followed by a stock solution of CuI (20 µL, 4.0 mM, 100 nmol, 0.004 eq.). The respective amount of hydroquinone was added as a solid followed by NaOAc buffer (348 µL, 250 mM, pH 5.5). The vials were sealed and mixed thoroughly (vortex), an aliquot was taken for GC-FID analysis and 70% aqueous TBHP solution (28.6 µL, 200 µmol, 8 eq.) was added in one portion at 22 °C. The vials were sealed and shaken in an orbital shaker (30 °C, 900 rpm, horizontal position). At the specified time points an aliquot of the reaction mixture (25.0 µL) was transferred into a separate Eppendorf vial (1.5 mL) with standard solution (200 µL, 6.25 µM 4'-methoxyacetophenone in EtOAc), the vial was vortexed (60 s) and centrifuged. The phases were separated and the organic layer was dried over Na_2SO_4 and analyzed by GC-FID.

Effect of double addition of reagents on the HQ-mediated, Cu-catalyzed trifluoromethylation

An Eppendorf vial (1.5 mL) was charged with a freshly prepared DMSO stock solution (63 µL) of acetosyringone (2.5 mg, 12.5 µmol, 1 eq.), $Zn(SO_2CF_3)_2 \cdot 2\,H_2O$ (4.2 mg, 12.5 µmol, 1 eq.) and hydroquinone (0.35 mg, 3.13 µmol, 0.25 eq.) followed by a stock solution of CuI (10 µL, 4.0 mM, 50 nmol, 0.004 eq.). NaOAc buffer (174 µL, 250 mM, pH 5.5) was added, the vial was sealed and mixed thoroughly (vortex), an aliquot was taken for GC-FID analysis and 70% aqueous TBHP solution (7.2 µL, 50.0 µmol, 4 eq.) was added in one portion at 22 °C. The vial was sealed and shaken in an orbital shaker (30 °C, 900 rpm, horizontal position) for 15 min. The vial was opened, $Zn(SO_2CF_3)_2 \cdot 2\,H_2O$ (4.2 mg, 12.5 µmol, 1 eq.) and hydroquinone (0.35 mg, 3.13 µmol, 0.25 eq.) were added as solids followed by a 70% aqueous TBHP solution (7.2 µL, 50.0 µmol, 4 eq.). The vial was sealed and shaken in an orbital shaker (30 °C, 900 rpm, horizontal position) for 24 h. An aliquot of the reaction mixture (25.0 µL) was transferred into a separate Eppendorf vial (1.5 mL) with standard solution (200 µL, 6.25 µM 4'-methoxyacetophenone in EtOAc), the vial was vortexed (60 s) and centrifuged. The phases were separated and the organic layer was dried over Na_2SO_4 and analyzed by GC-FID.

HQ-mediated Cu-catalyzed trifluoromethylation on preparative scale

A 15 mL Sarstedt tube was charged with the phenol/anisole (200 µmol, 1 eq.), hydroquinone (100 µmol, 0.5 eq.), $Zn(SO_2CF_3) \cdot 2\,H_2O$ (400 µmol, 2 eq.) and 848 µL DMSO. The mixture was vortexed and placed into an ultrasonic bath until a homogenous solution was obtained. CuI (800 nmol, 0.004 eq.) was added as a stock solution (160 µL, 1.5 mg CuI in 2.00 mL DMSO) followed by NaOAc buffer (2.8 mL, 250 mM, pH 5.5) and the mixture was thoroughly mixed. TBHP (70% in H_2O, 1.60 mmol, 8 eq.) was added in one portion, the tube was sealed, horizontally placed into an orbital shaker and was shaken at 500 rpm at 30 °C for the time specified in the respective procedures. EtOAc (6 mL) was added and the mixture was

thoroughly mixed (vortex). The phases were separated and the aqueous layer was extracted with EtOAc (4 x 6 mL). The combined organic phase was dried over Na_2SO_4, filtered and the solvent was removed on the rotary evaporator. The product was purified by preparative reversed-phase HPLC using the gradients specified in the respective procedures.

SI-2. Experimental Spectra

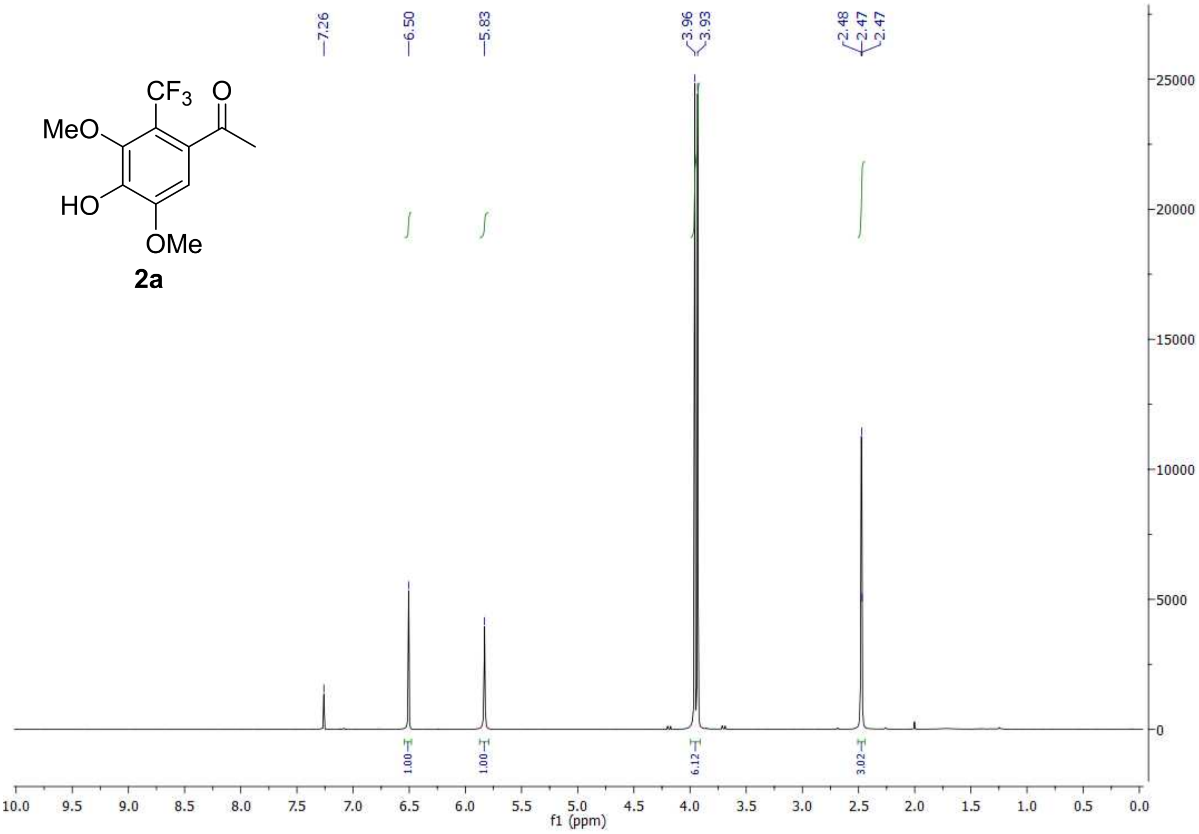

Figure SI-5: ^{1}H-NMR spectrum of 1-(4-hydroxy-3,5-dimethoxy-2-(trifluoromethyl)phenyl)ethan-1-one (**2a**).

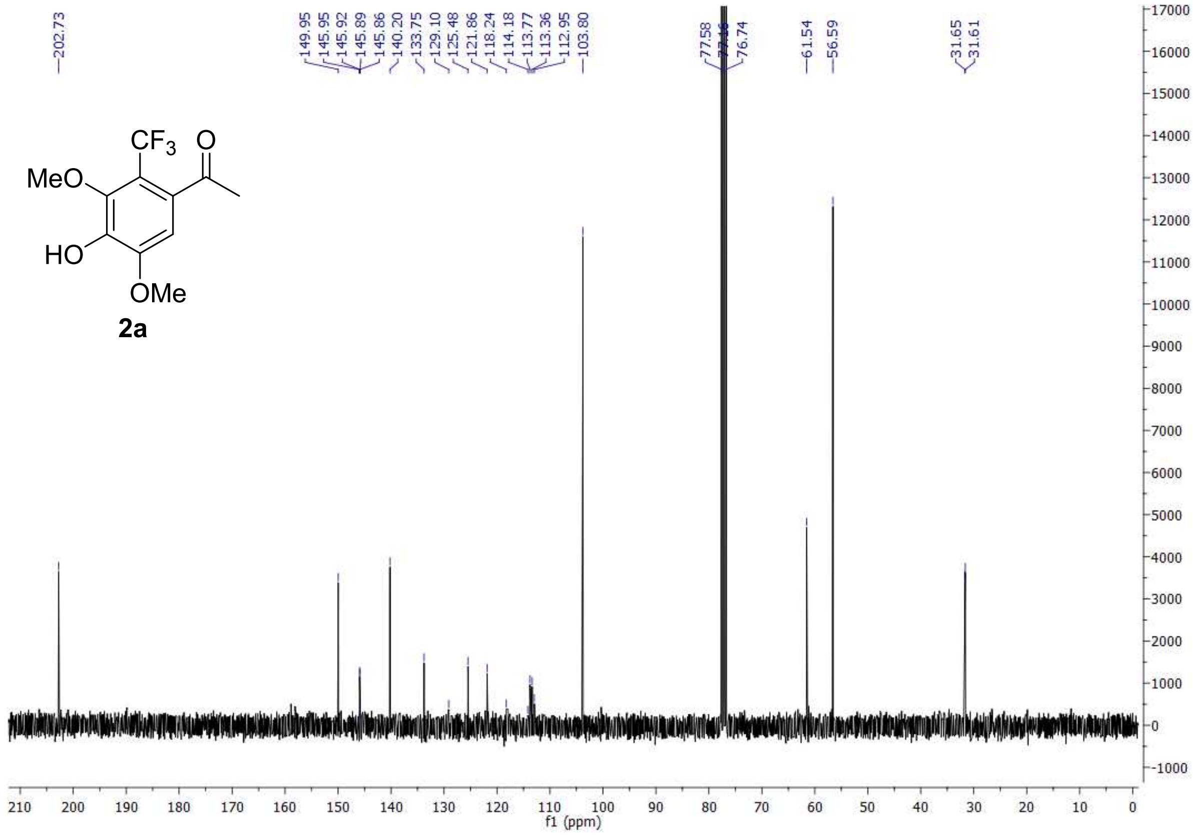

Figure SI-6: ^{13}C-NMR spectrum of 1-(4-hydroxy-3,5-dimethoxy-2-(trifluoromethyl)phenyl)ethan-1-one (**2a**).

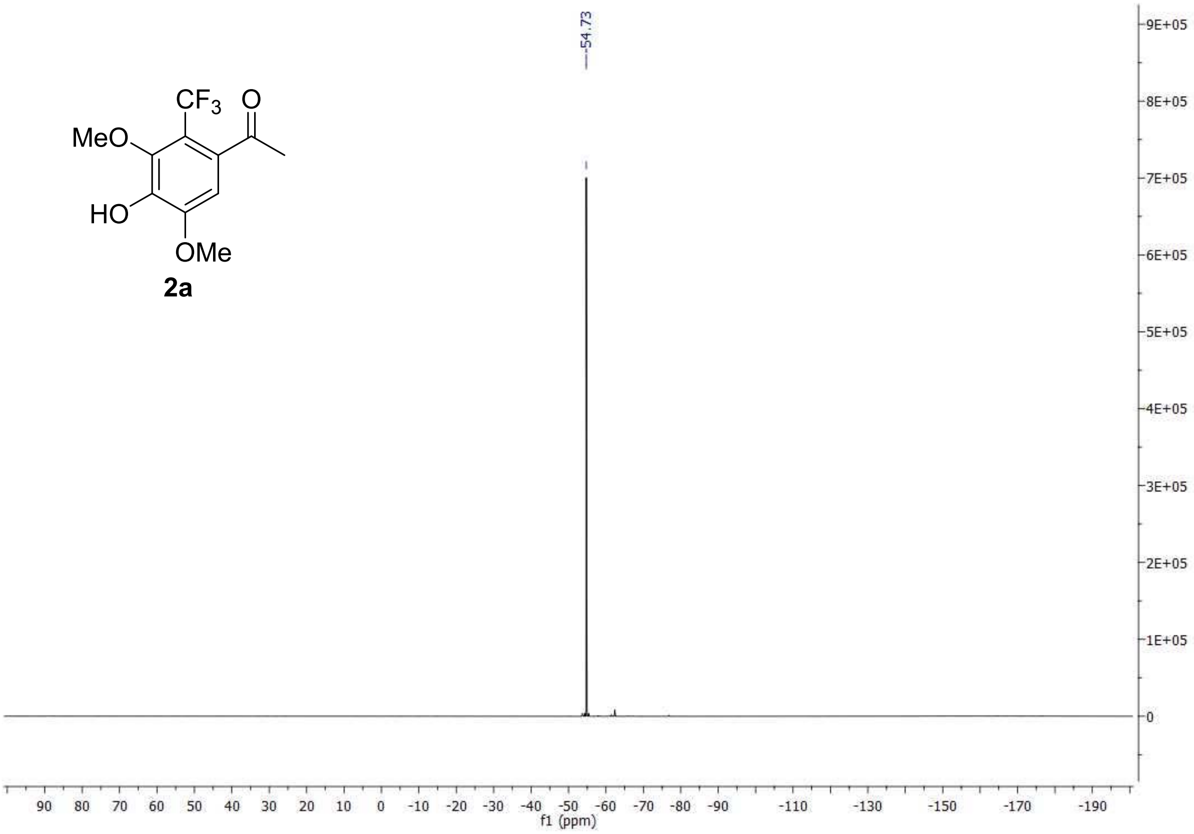

Figure SI-7: ^{19}F-NMR spectrum of 1-(4-hydroxy-3,5-dimethoxy-2-(trifluoromethyl)phenyl)ethan-1-one (**2a**).

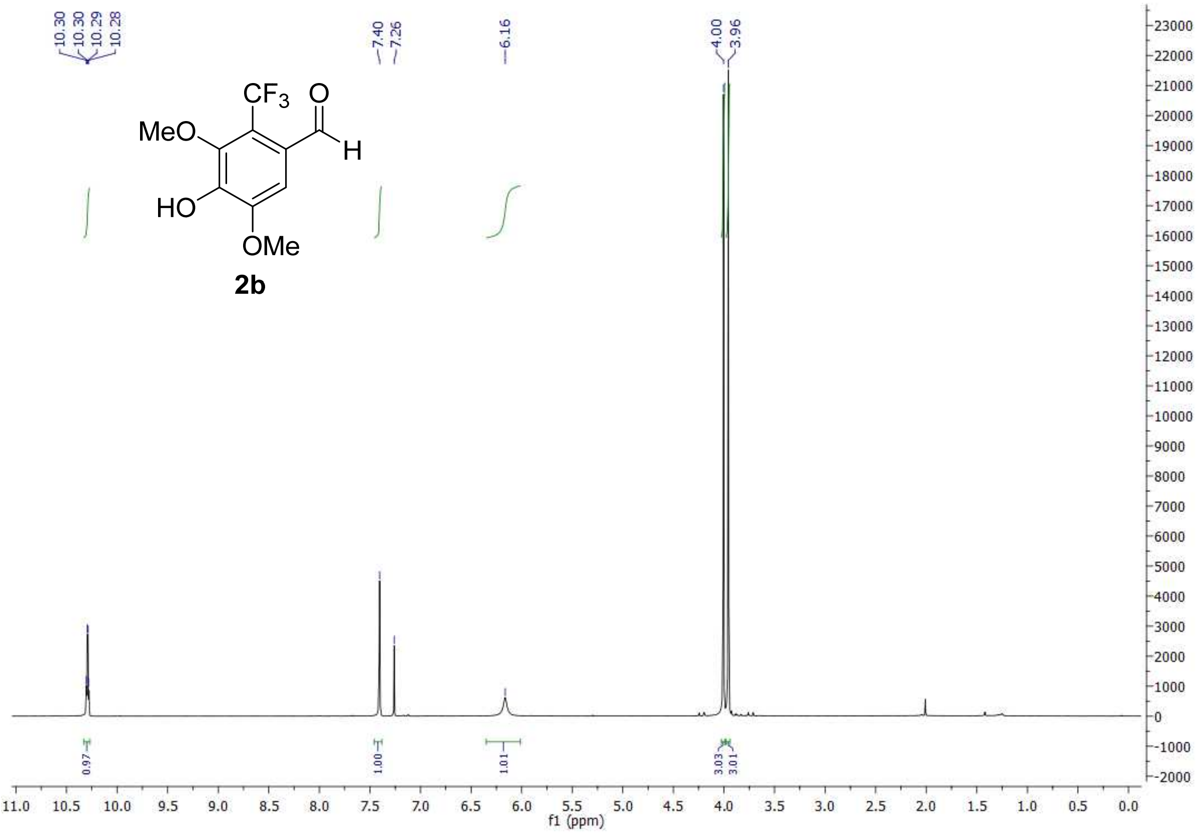

Figure SI-8: ^{1}H-NMR spectrum of 4-hydroxy-3,5-dimethoxy-2-(trifluoromethyl)benzaldehyde (**2b**).

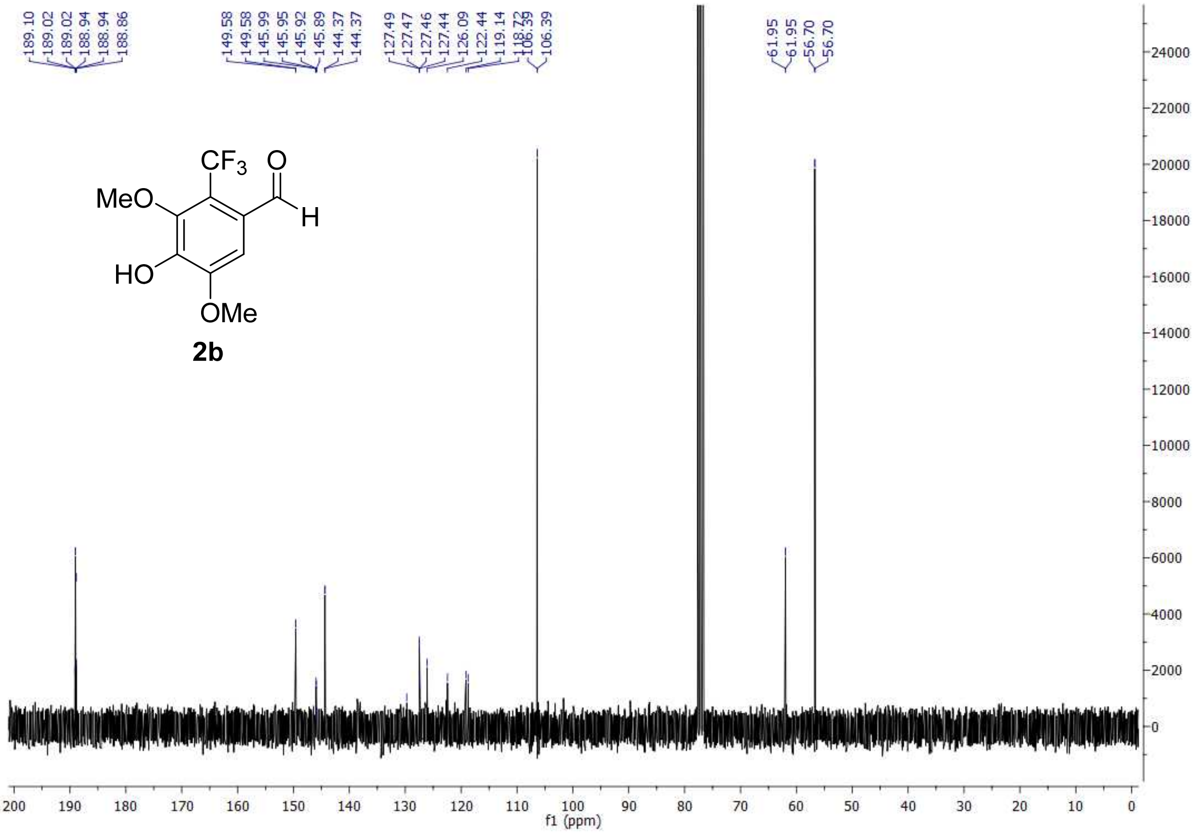

Figure SI-9: ^{13}C-NMR spectrum of 4-hydroxy-3,5-dimethoxy-2-(trifluoromethyl)benzaldehyde (**2b**).

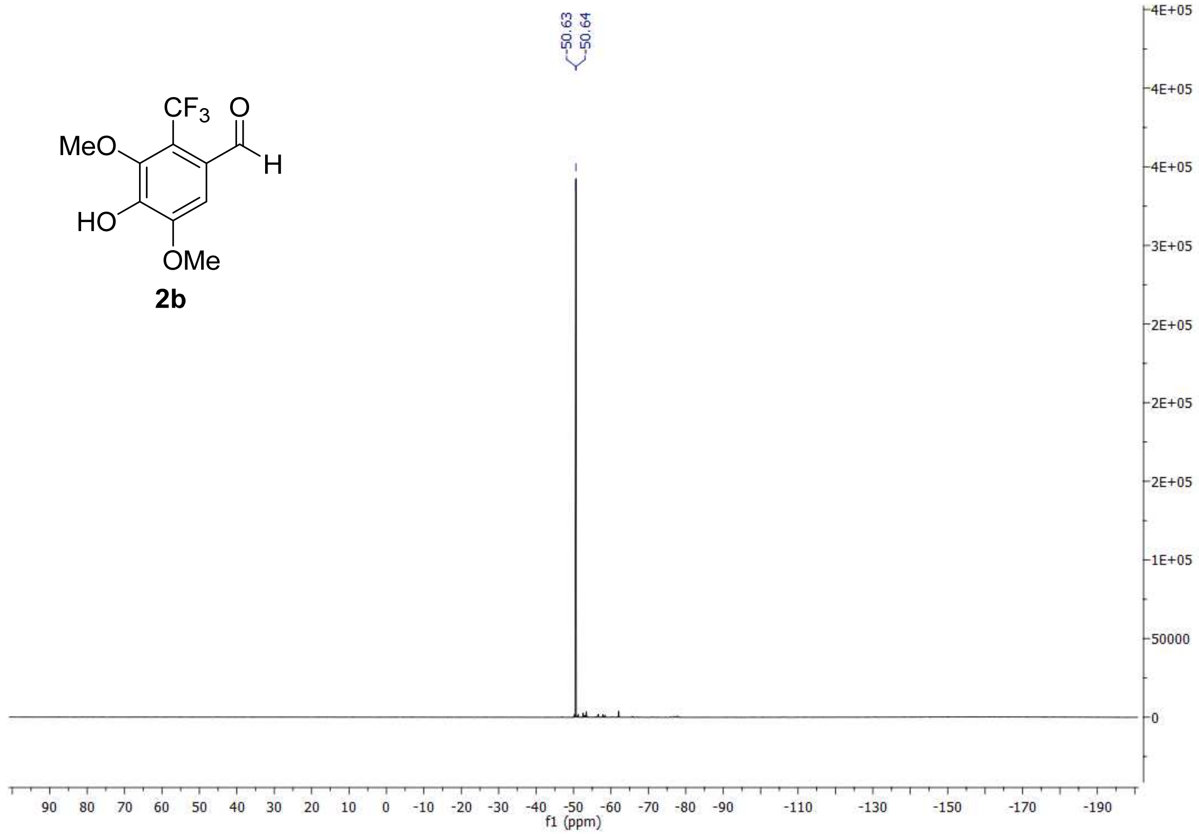

Figure SI-10: ^{19}F-NMR spectrum of 4-hydroxy-3,5-dimethoxy-2-(trifluoromethyl)benzaldehyde (**2b**).

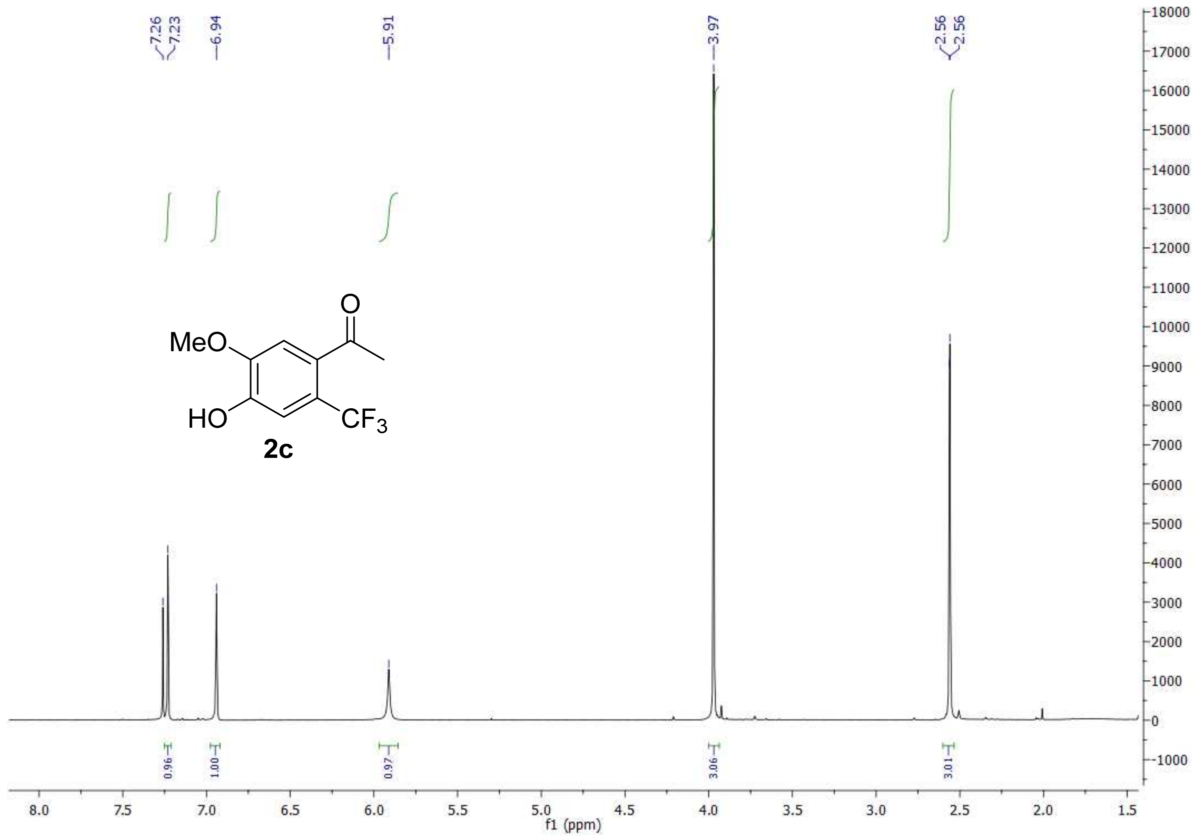

Figure SI-11: ^{1}H-NMR spectrum of 1-(4-hydroxy-5-methoxy-2-(trifluoromethyl)phenyl)ethan-1-one (**2c**).

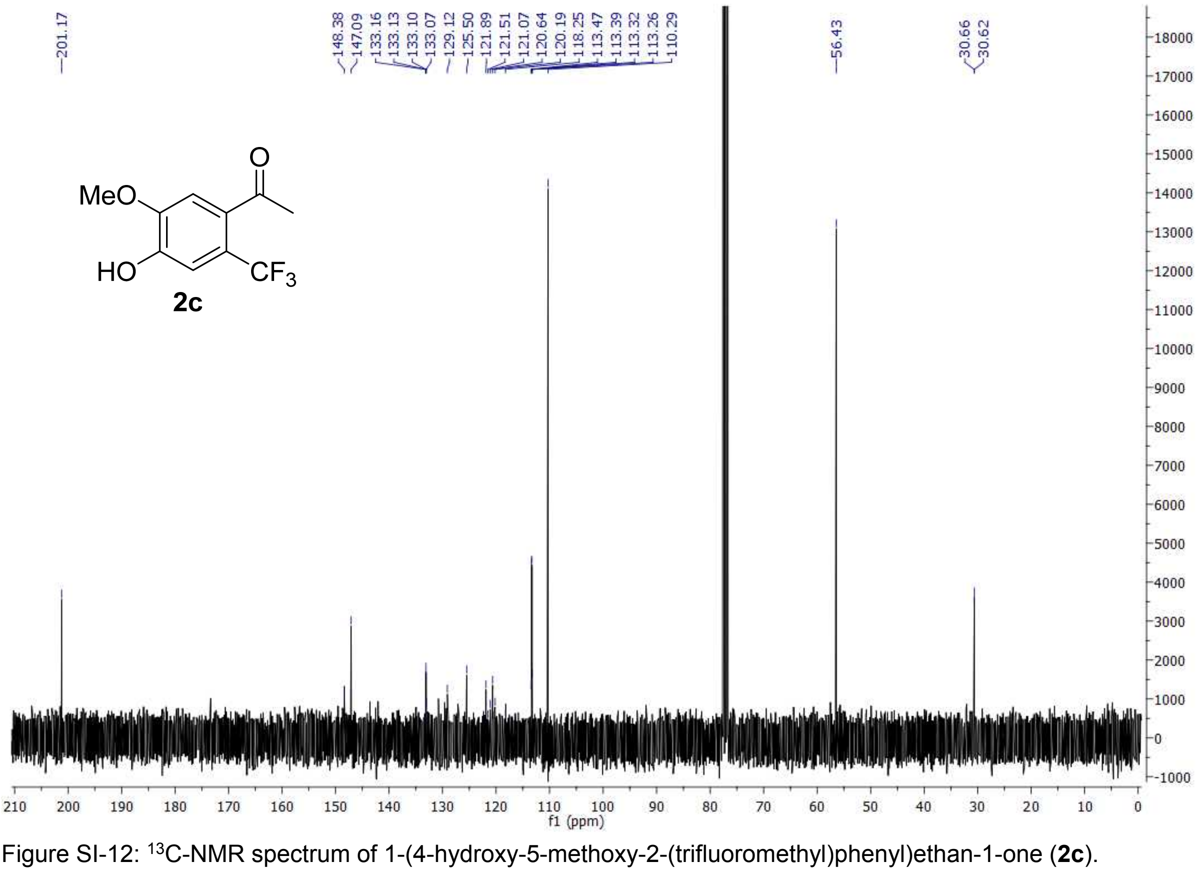

Figure SI-12: ^{13}C-NMR spectrum of 1-(4-hydroxy-5-methoxy-2-(trifluoromethyl)phenyl)ethan-1-one (**2c**).

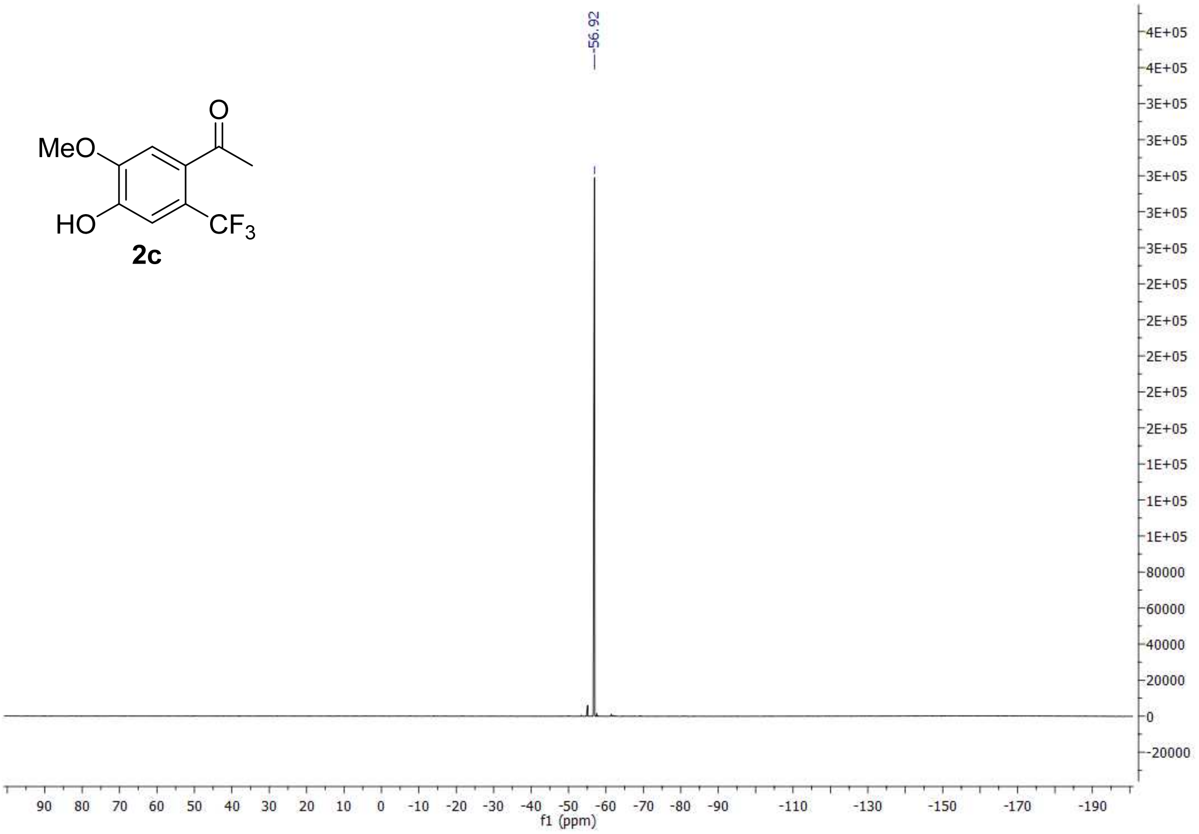

Figure SI-13: ^{19}F-NMR spectrum of 1-(4-hydroxy-3,5-dimethoxy-2-(trifluoromethyl)phenyl)ethan-1-one (**2c**)

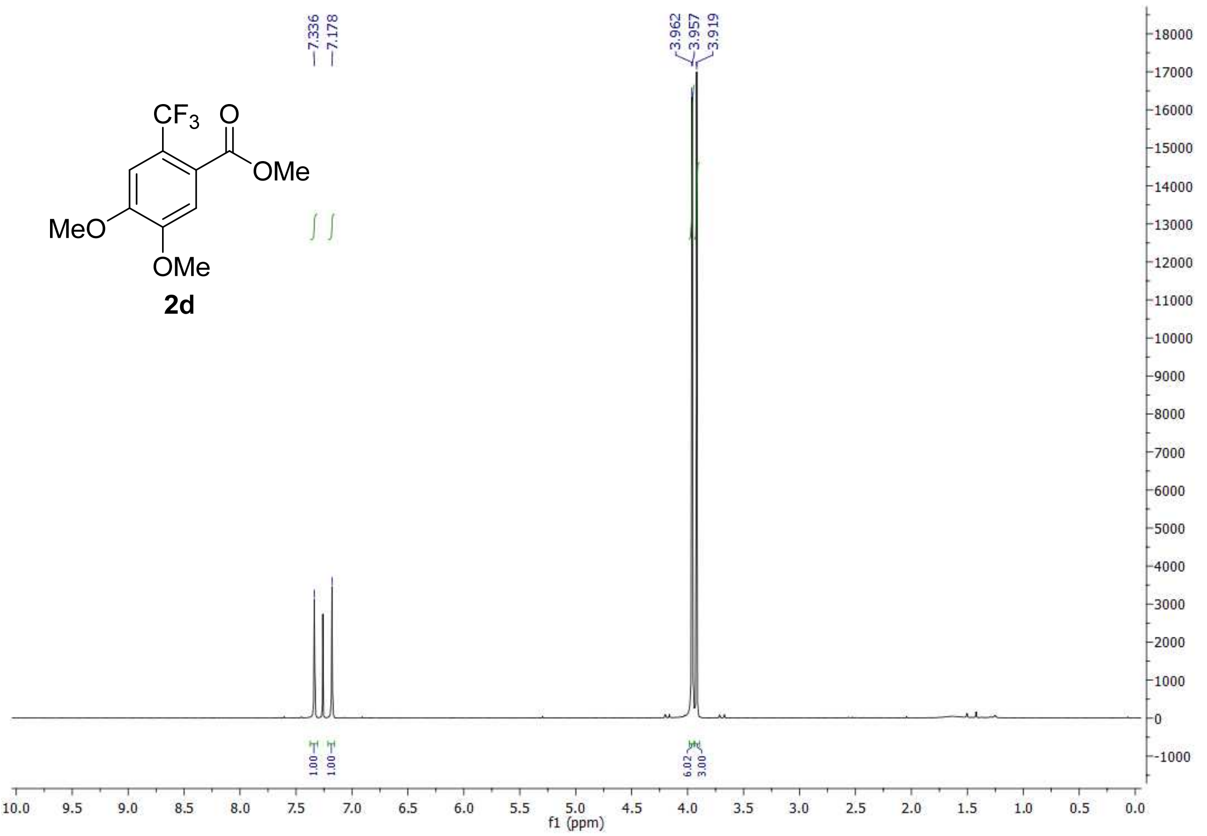

Figure SI-14: ^{1}H-NMR spectrum of methyl 4,5-dimethoxy-2-(trifluoromethyl)benzoate (**2d**).

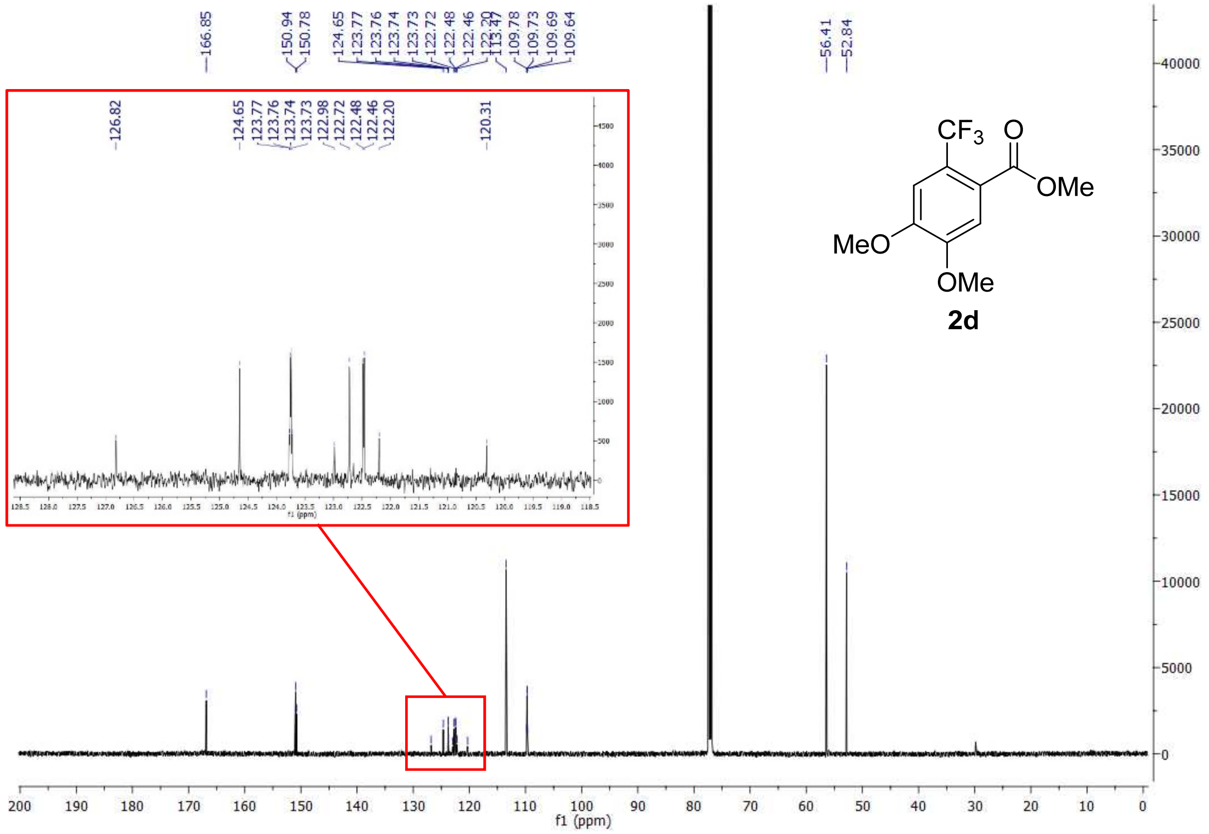

Figure SI-15: ^{13}C-NMR spectrum of methyl 4,5-dimethoxy-2-(trifluoromethyl)benzoate (**2d**).

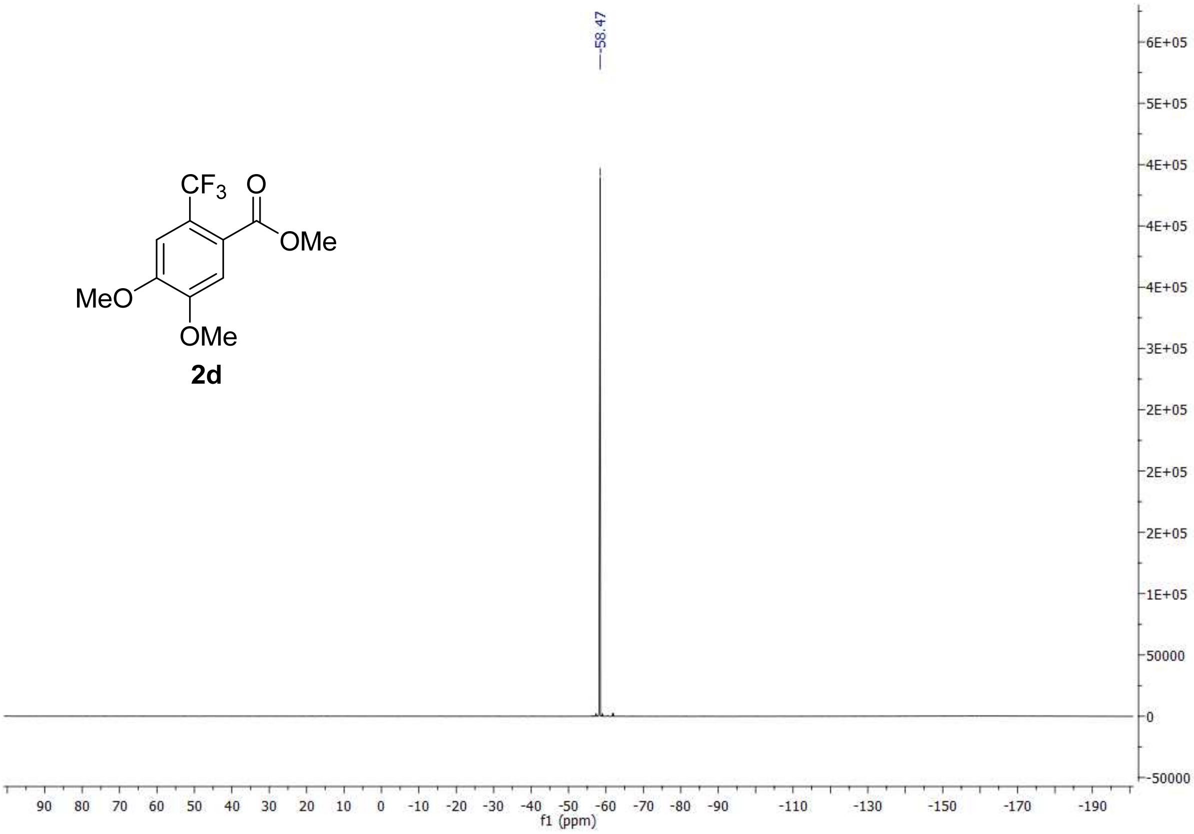

Figure SI-16: ^{19}F-NMR spectrum of methyl 4,5-dimethoxy-2-(trifluoromethyl)benzoate (**2d**).

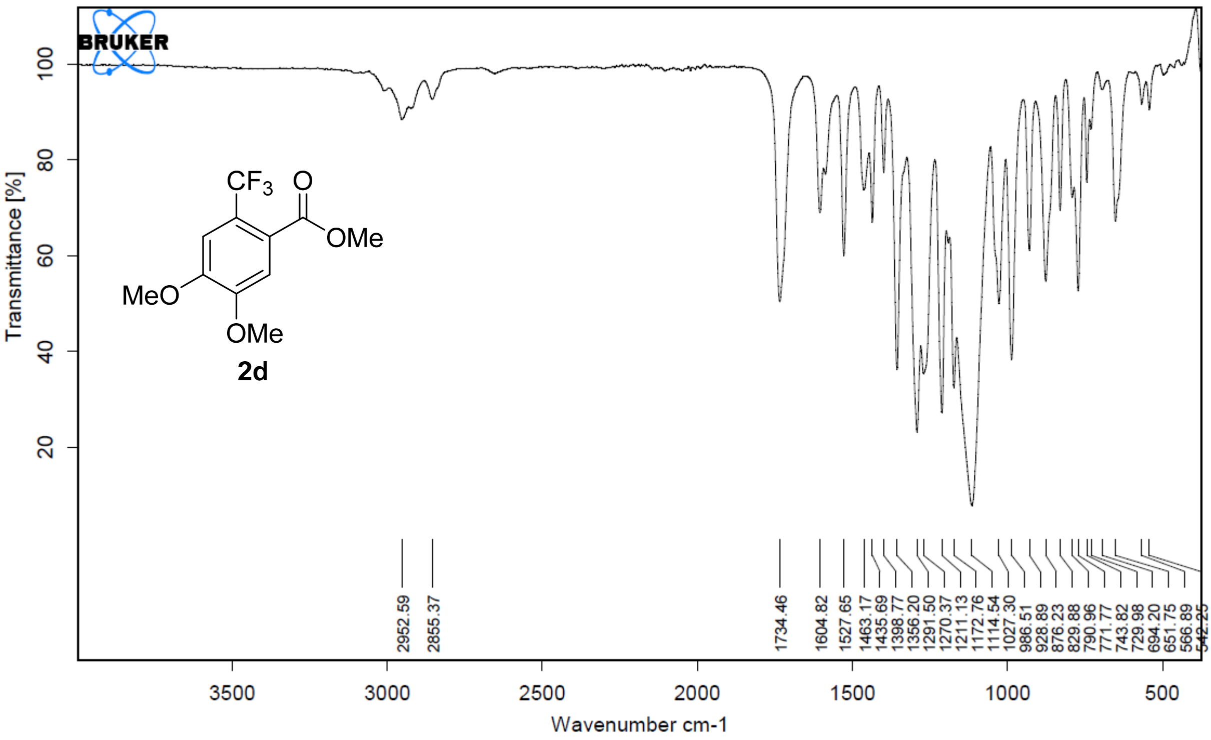

C:\Programme\OPUS_65\MEAS\JPL_3-407-f132b133.0	JPL_3-407-f132b133	Instrument type and / or accessory	18/05/2018

Figure SI-17: ATR-IR spectrum of methyl 4,5-dimethoxy-2-(trifluoromethyl)benzoate (**2d**).

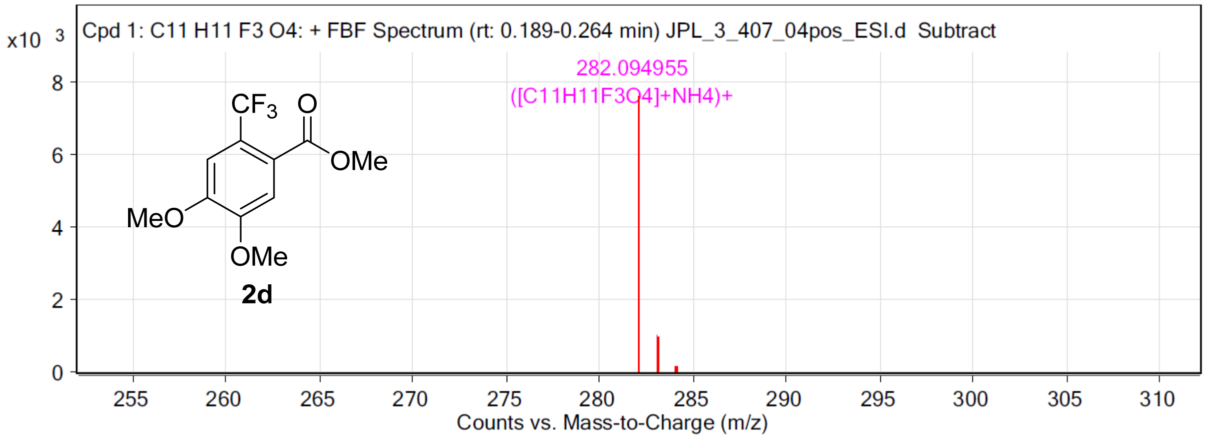

Figure SI-18: HR-TOF-MS spectrum of methyl 4,5-dimethoxy-2-(trifluoromethyl)benzoate (**2d**).

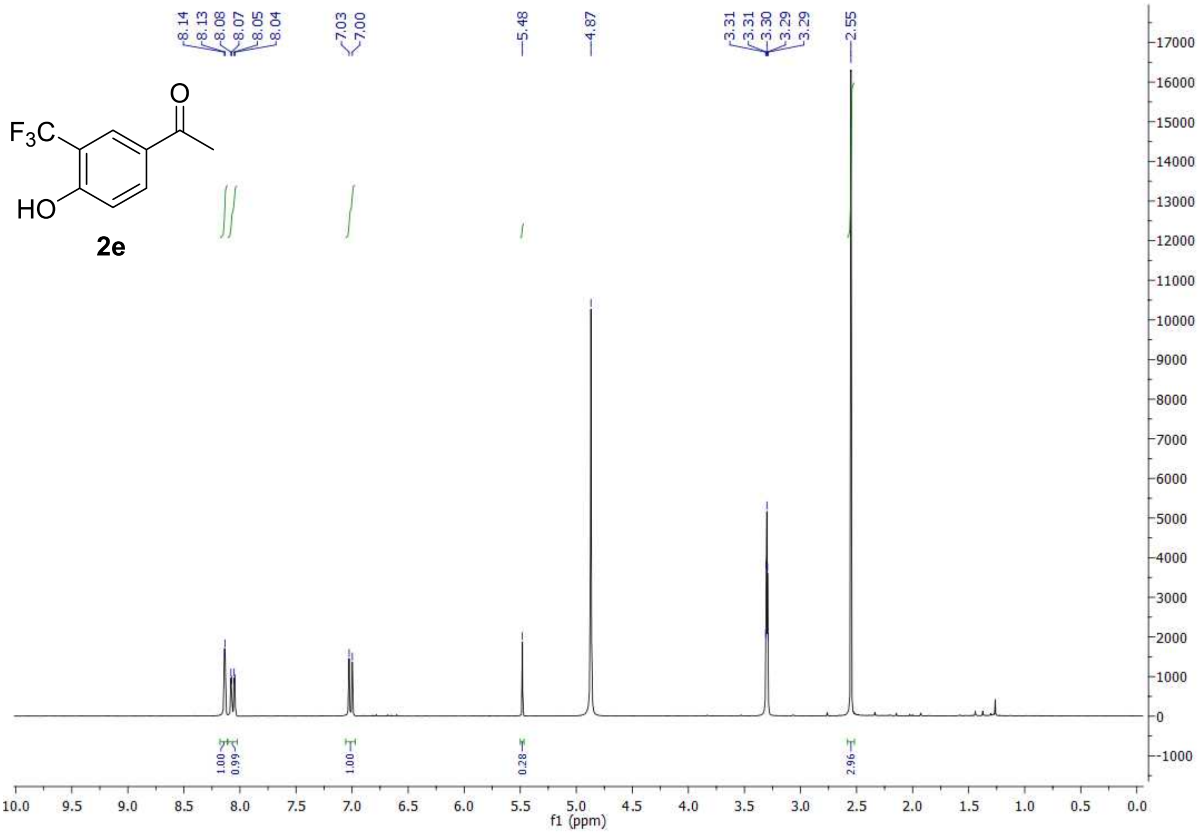

Figure SI-19: ^{1}H-NMR spectrum of 1-(4-hydroxy-3-(trifluoromethyl)phenyl)ethan-1-one (**2e**).

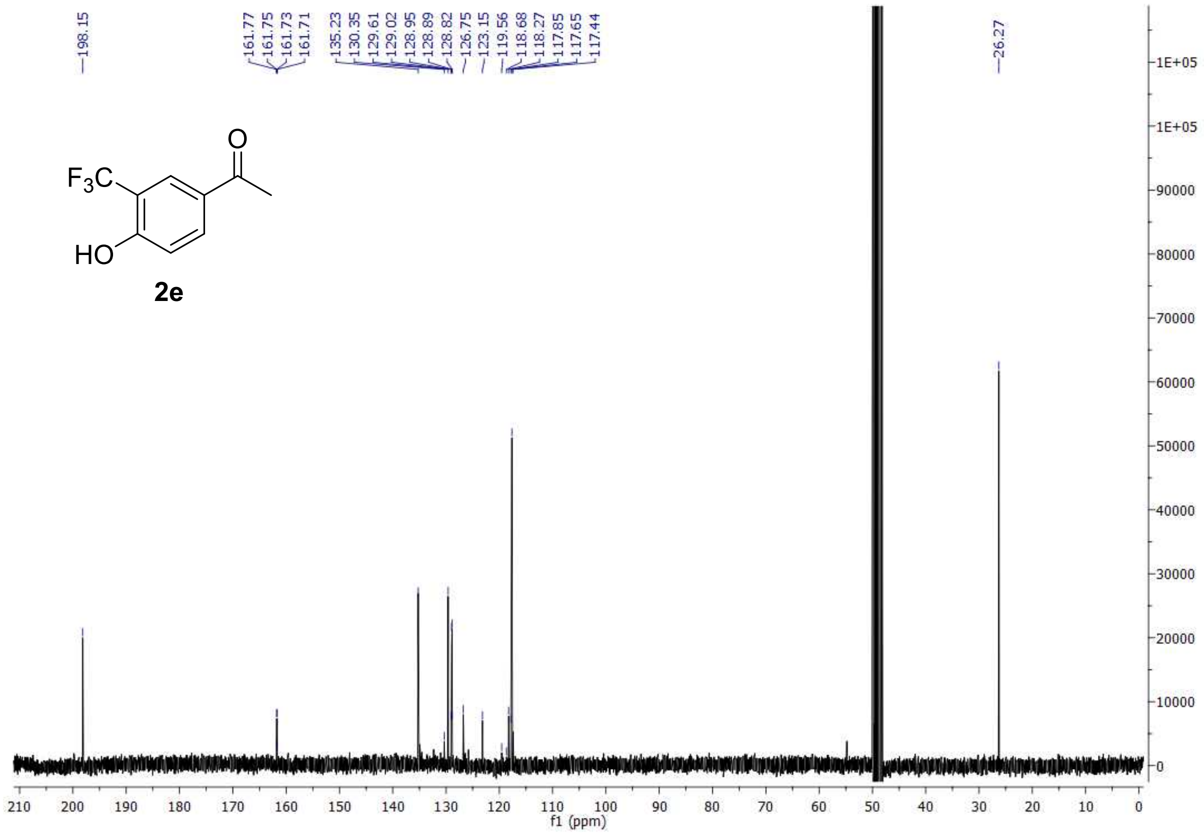

Figure SI-20: ^{13}C-NMR spectrum of 1-(4-hydroxy-3-(trifluoromethyl)phenyl)ethan-1-one (**2e**).

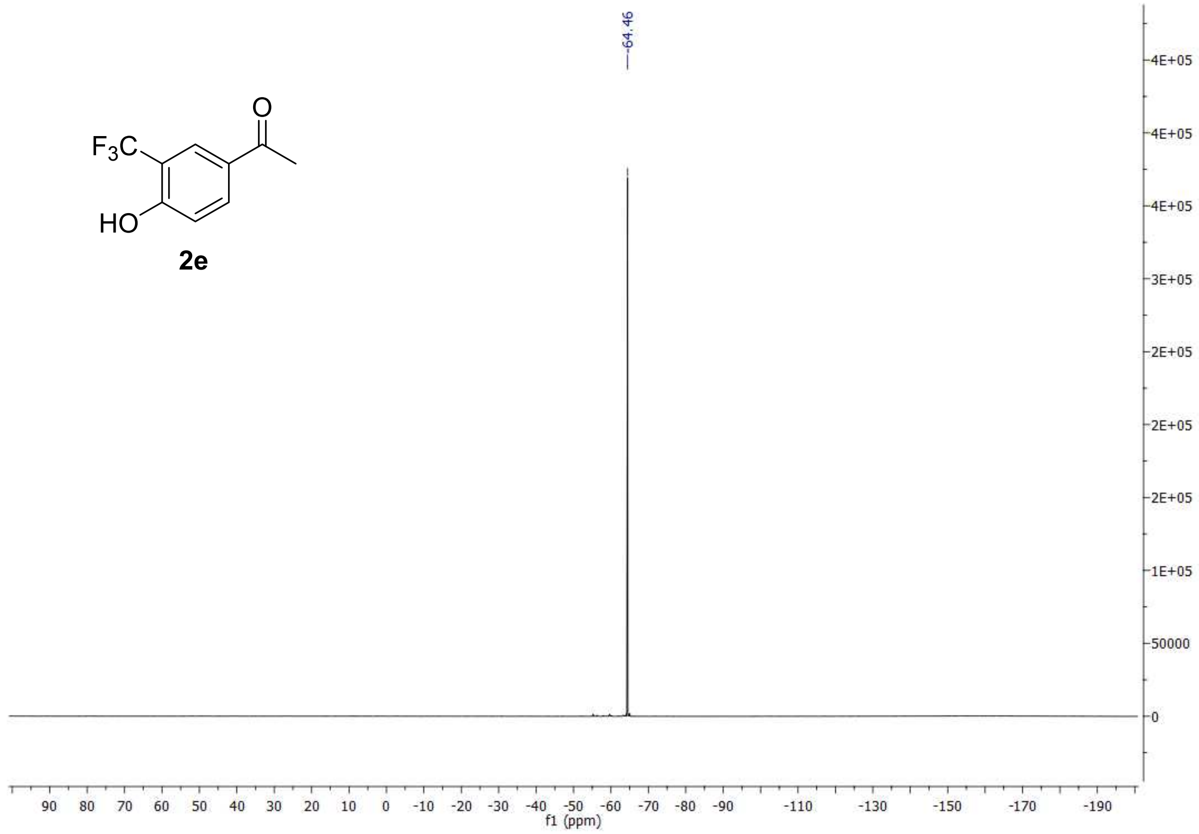

Figure SI-21: ^{19}F-NMR spectrum of 1-(4-hydroxy-3-(trifluoromethyl)phenyl)ethan-1-one (**2e**).

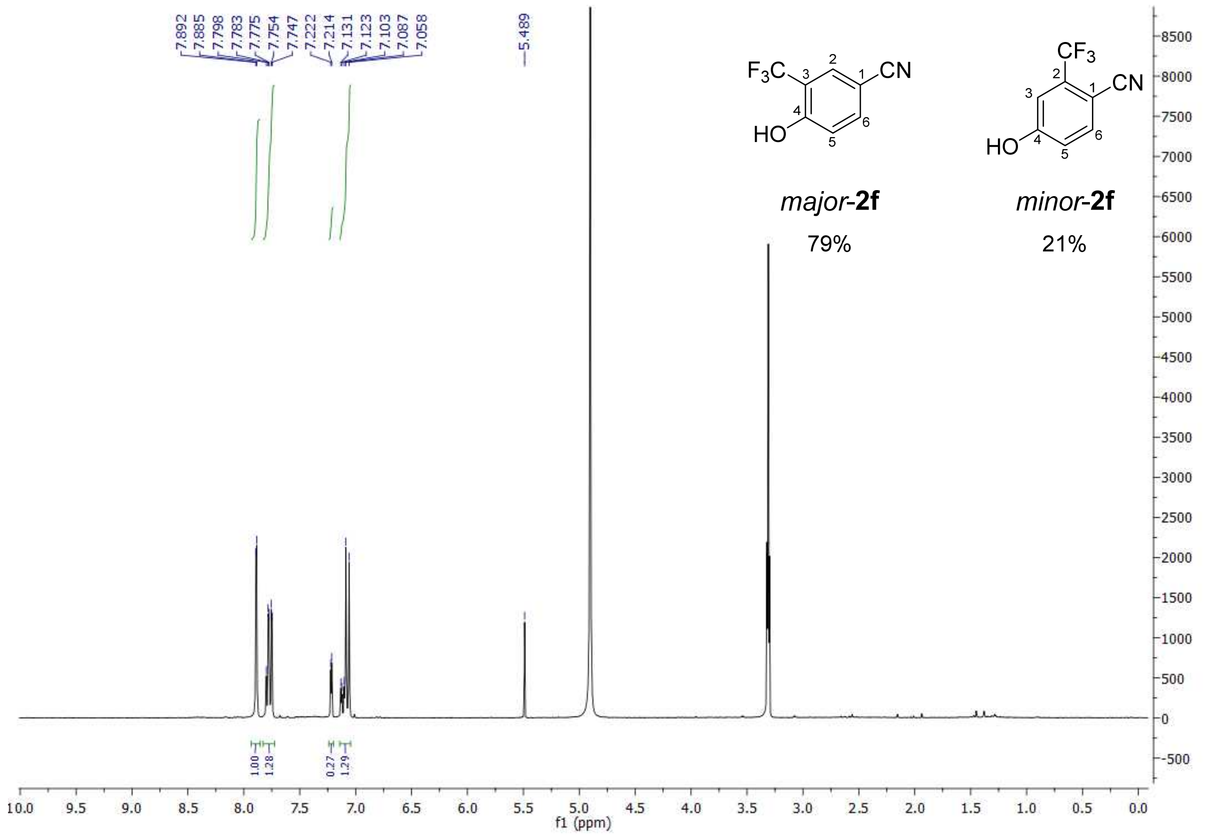

Figure SI-22: ^{1}H-NMR spectrum (MeOD-d$_4$) of the trifluoromethylation products of 4-hydroxybenzonitrile (**1f**).

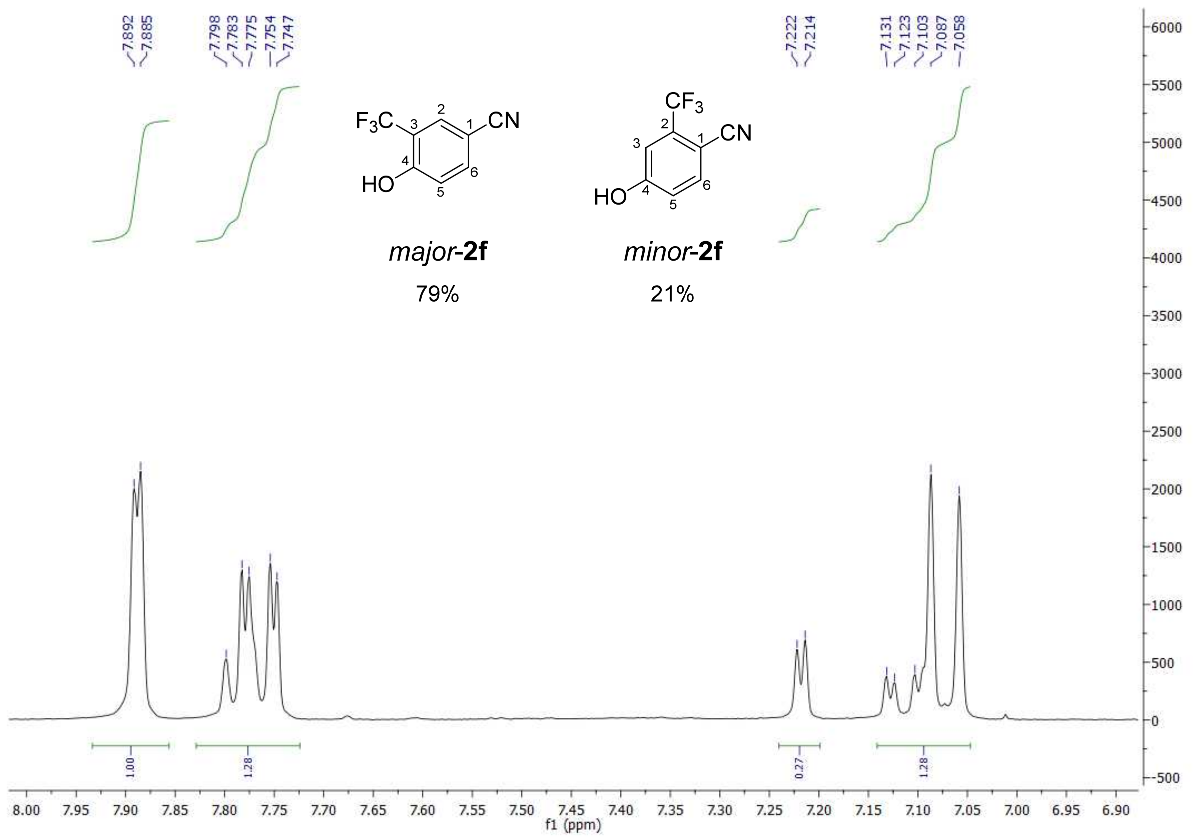

Figure SI-23: Aromatic region of the ^{1}H-NMR spectrum (MeOD-d4) of the trifluoromethylation products of 4-hydroxybenzonitrile (**1f**). Ratio(*major*/*minor*) according to ^{1}H-NMR: 79:21.

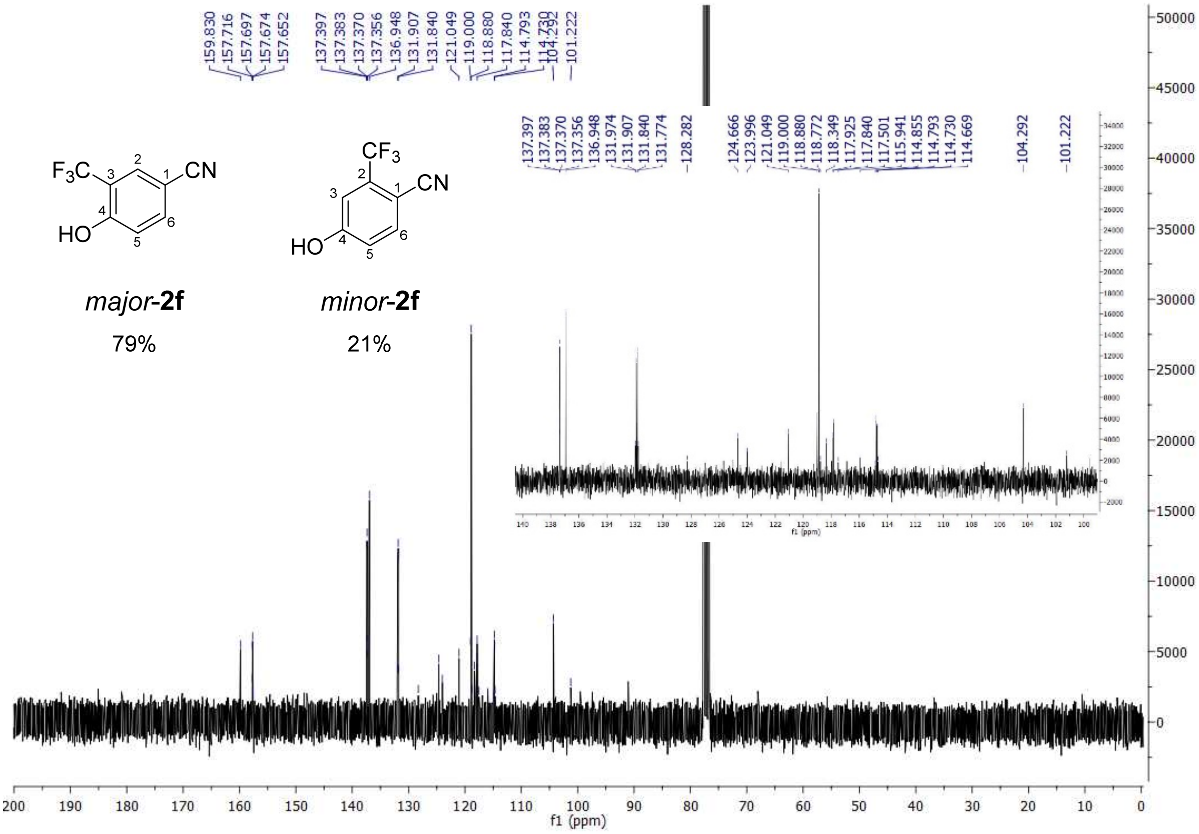

Figure SI-24: ^{13}C-NMR spectrum ($CDCl_3$) of the trifluoromethylation products of 4-hydroxybenzonitrile (**1f**).

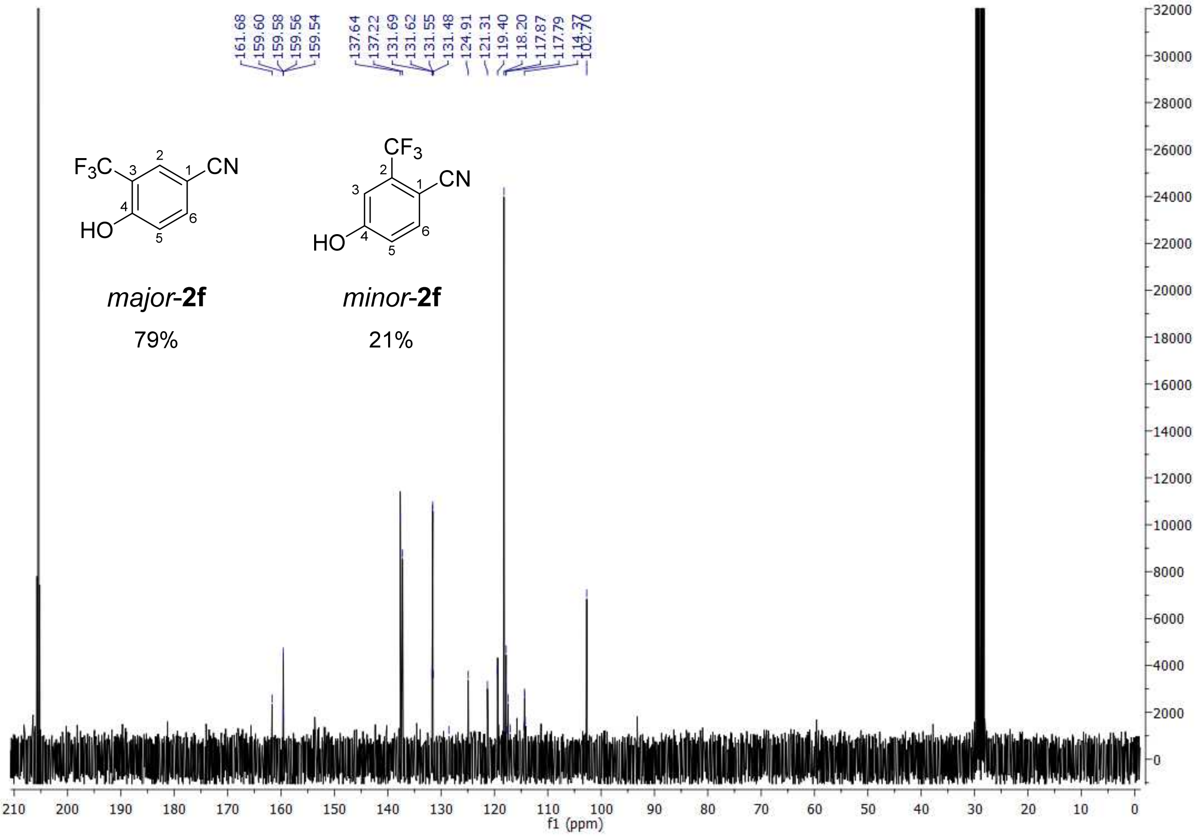

Figure SI-25: ^{13}C-NMR spectrum (acetone-d_6) of the trifluoromethylation products of 4-hydroxybenzonitrile (**1f**).

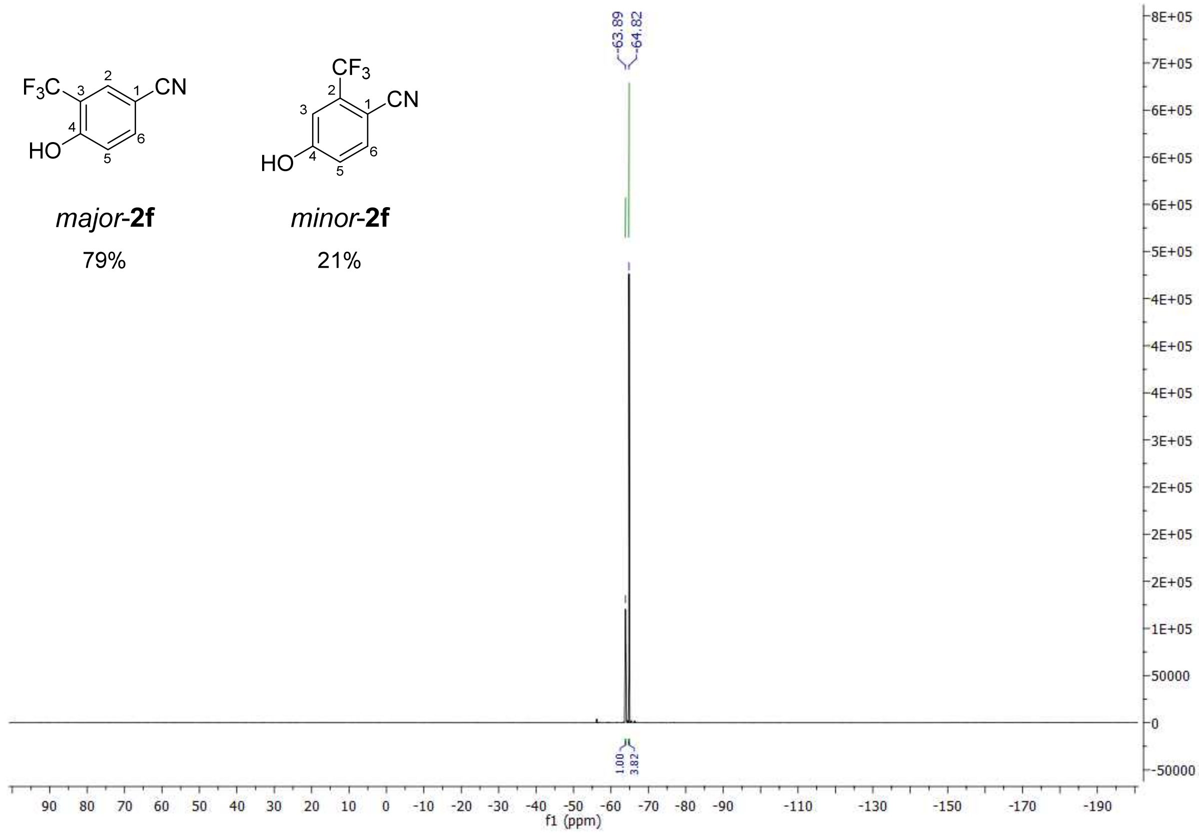

Figure SI-26: ^{19}F-NMR spectrum of the trifluoromethylation products of 4-hydroxybenzonitrile (**1f**). ^{19}F-NMR area ratio (major/minor) = 79:21.

SI-3. References

[1] Y.-Y. Wang, R. Lu, L. Xiao, Y.-M. Du, T. Miyakoshi, C.-L. Chen, C. J. Knill, J. F. Kennedy, *Int. J. Biol. Macromol.* **2010**, *47*, 488–495.

[2] J. P. Kallio, S. Auer, J. Jänis, M. Andberg, K. Kruus, J. Rouvinen, A. Koivula, N. Hakulinen, *J. Mol. Biol.* **2009**, *392*, 895–909.

[3] R. C. Simon, E. Busto, N. Richter, V. Resch, K. N. Houk, W. Kroutil, *Nat. Commun.* **2016**, *7*, 13323.
